# The association between a pro-inflammatory diet and brain age in middle-aged and older adults

**DOI:** 10.1007/s10654-025-01318-6

**Published:** 2025-10-27

**Authors:** Michelle M. Dunk, Huijie Huang, Jiao Wang, Abigail Dove, Sakura Sakakibara, Jie Guo, Adrián Carballo-Casla, David A. Bennett, Weili Xu

**Affiliations:** 1 Aging Research Center, Department of Neurobiology, Care Sciences and Society, Karolinska Institutet, Tomtebodavägen 18A, Floor 10, 171 65 Stockholm, Sweden; 2 Division of Neurogeriatrics, Department of Neurobiology, Care Sciences and Society, Karolinska Institutet, Stockholm, Sweden; 3 Department of Epidemiology and Biostatistics, School of Public Health, Tianjin Medical University, Tianjin, China; 4 National Clinical Research Center for Geriatrics, West China Hospital, Sichuan University, Chengdu, China; 5 Aging Research Center, Department of Neurobiology, Care Sciences and Society, Karolinska Institutet, Stockholm, Sweden; 6 Department of Nutrition and Health, China Agricultural University, Beijing, China; 7 CIBER of Epidemiology and Public Health (CIBERESP), Madrid, Spain; 8 Rush Alzheimer’s Disease Center, Rush University Medical Center, Chicago, IL USA; 9 Tianjin Key Laboratory of Environment, Nutrition and Public Health, Tianjin, China; 10 Center for International Collaborative Research On Environment, Nutrition, and Public Health, Tianjin, China

**Keywords:** Dietary inflammatory index, Anti-inflammatory diet, Brain magnetic resonance imaging, Machine learning, Brain age gap, UK Biobank

## Abstract

Pro-inflammatory diets are associated with cognitive decline and dementia, but their impact on brain aging is unclear. We investigated the association between a pro-inflammatory diet and brain age, taking into account age, genetic risk for dementia, and systemic inflammation. UK Biobank participants (N = 21,473) aged 40–70 years and free of neurological disorders were included. The Dietary Inflammatory Index (DII) was calculated from participants’ average intake of 31 nutrients, assessed up to five times via 24-h recalls. Participants were categorized into four DII groups (group 1, anti-inflammatory, DII < −2; group 2, DII −2 to < 0; group 3, DII 0 to < 2; group 4, DII ≥ 2), with group 4 reflecting the most pro-inflammatory diet. Brain age was estimated using a machine learning model based on 1079 structural and functional MRI measures, obtained approximately 9 years after baseline. We calculated brain age gap (BAG; brain age minus chronological age), where BAG > 0 reflects a biologically older brain than chronological age. An Alzheimer’s disease polygenic risk score (PRS_AD_), *APOE*4 status, and a composite score of systemic inflammation (INFLA-score) were determined from baseline blood samples. More pro-inflammatory diets were associated with increasingly greater BAG, with advanced brain age by $\widehat{\upbeta }$=0.50 [95% CI 0.20, 0.80] years among those in group 4. There were no interactions between DII and age, PRS_AD_, or *APOE*4 in relation to BAG, but associations were stronger among adults ≥ 60 years. INFLA-score mediated 8% of the DII-BAG association. These findings suggest that a pro-inflammatory diet may accelerate brain aging, especially in older adults.

**Supplementary Information:**

The online version contains supplementary material available at 10.1007/s10654-025-01318-6.

## Introduction

There is growing evidence that chronic systemic inflammation contributes to the development of neurodegenerative diseases [1, 2]. Inflammatory biomarkers such as C-reactive protein (CRP) and interleukin 6 (IL-6) tend to increase with age [3], and elevated levels of these biomarkers are associated with greater risk of cognitive decline and dementia [2, 4, 5]. Chronic systemic inflammation may even contribute to neuropathogenesis of Alzheimer’s disease (AD) [2], the most common form of dementia [6].

The literature indicates that age-related systemic inflammation may be modifiable through diet [7]. For example, consumption of the pro-inflammatory Western dietary pattern, which is high in red and processed meat, high-fat dairy, eggs, refined grains, and processed foods, is associated with higher levels of inflammatory biomarkers [7, 8]. In contrast, anti-inflammatory dietary patterns which are higher in minimally processed plant foods such as vegetables, fruits, whole grains, and legumes tend to be associated with lower levels of inflammatory biomarkers [8, 9]. Considering the involvement of systemic inflammation in neurodegeneration, these findings point to the possibility that dietary modification of systemic inflammation could in turn support brain health.

Pro-inflammatory diets have in fact been associated with increased risk for cognitive impairment and dementia [10–13], as well as several individual markers of brain aging using magnetic resonance imaging (MRI) [14]. However, whether pro-inflammatory diets are associated with a comprehensive measure of overall brain aging has not been examined. Deterioration of brain structure and function beyond normal aging is a risk factor for dementia [15, 16], so clarifying the impact of pro-inflammatory diets on brain aging could provide critical insights into disease pathogenesis. It is also unclear whether older adults and those at genetic risk for dementia may be more susceptible to harmful effects of pro-inflammatory diets. Given that age and genetics are two of the greatest risk factors for dementia [6], clarifying their involvement in the relationship between diet and brain aging could shed light on who may benefit most from dietary interventions.

The purpose of the current study was to investigate the relationship between a pro-inflammatory diet and a rigorous measure of global brain age in the UK Biobank, a large-scale prospective study of UK adults. Through the integration of MRI and machine learning, brain age estimation can identify deviation from the typical aging trajectory well before the occurrence of clinically detectable symptoms [17]. Brain age estimation therefore offers an invaluable tool to study preventative strategies for neurodegeneration [17]. Our aims were threefold: (1) to investigate the relationship between a pro-inflammatory diet and brain age gap (BAG), an indicator of advanced brain aging; (2) to examine whether this association differs by age and genetic risk for dementia; and (3) to determine whether inflammatory markers mediate this association.

## Methods

### Study population

**Fig. 1:**
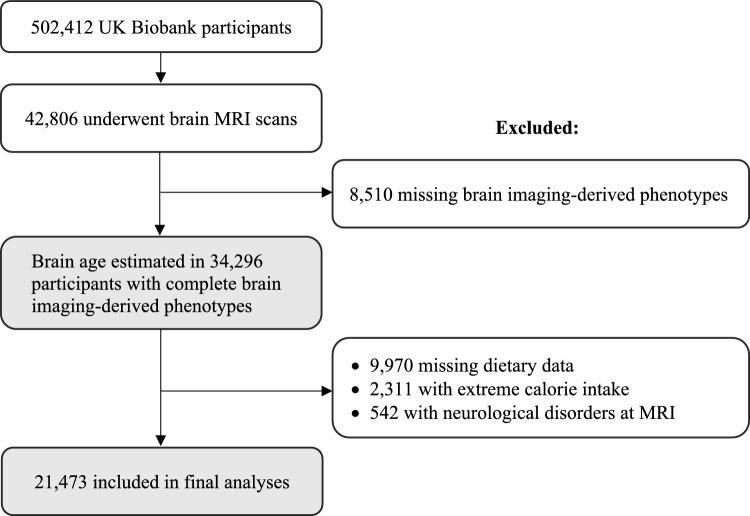
Flowchart of participant inclusion. *Abbreviations*: MRI, magnetic resonance imaging

The UK Biobank includes over 500,000 adults ages 40–70 years from the United Kingdom [18]. Participants completed assessment visits at one of 22 centers across the UK between 2006 and 2010. Sociodemographic, lifestyle, and health-related information was collected from a self-completed touchscreen questionnaire and computer-assisted interview [18, 19]. Physical and functional measurements and blood samples were also collected at baseline assessments [18]. A total of 42,806 participants underwent structural and functional brain MRI scans an average of 9 years after baseline (2014–2020), of whom 34,296 had complete MRI data. After further excluding 9,970 participants without dietary data, 2,311 who reported implausible energy intake in all of their dietary assessments (< 600 or > 3500 kcal/day for females; < 800 or > 4200 kcal/day for males), and 542 with neurological disorders at the time of the MRI scan (Supplementary Table S1), the current sample consisted of 21,473 participants (Fig. 1). All participants provided written informed consent, and the UK Biobank was approved by the North West Centre for Research Ethics Committee of the National Health Services (NHS) (11/NW/0382). Approval and data access for the current study was obtained through application 67048.

### Dietary assessment

Self-reported dietary information was collected using the Oxford WebQ, a web-based 24-h dietary assessment administered via touchscreen [20]. The Oxford WebQ inquires about consumption of 206 types of foods and 32 types of drinks over the previous 24 h [20]. Participants’ reported consumption frequency of each food or drink was multiplied by a standard portion size and the nutrient composition of that item to calculate the mean daily intake of nutrients [21]. The Oxford WebQ provides similar estimates of nutrient intake compared to interviewer-based 24-h dietary recall [22], and validation information has previously been published [23]. Participants completed at least one assessment during the baseline visit (2009–2010, n = 5055) or online between February 2011 and June 2012 (online cycle 1, February 2011 to April 2011, n = 11,698; online cycle 2, June 2011 to September 2011, n = 10,035; online cycle 3, October 2011 to December 2011, n = 12,775; online cycle 4, April 2012 to June 2012, n = 12,440) [20]. Assessments with extreme energy intake according to either the old or newer version of UK Biobank’s food composition tables were excluded [21]. Participants’ intake of each nutrient was averaged from all available dietary assessments, given that dietary intake from more than one Oxford WebQ is likely to more accurately reflect participants’ usual dietary habits [21].

### Dietary inflammatory index

We estimated the inflammatory potential of participants’ diets with the Dietary Inflammatory Index (DII) [24, 25]. The DII is a literature-derived, population-based index of 45 food parameters (including nutrients, bioactive compounds, whole foods, and spices) based on their associations with six inflammatory biomarkers (IL-1β, IL-4, IL-6, IL-10, tumor necrosis factor[TNF]-α, and CRP) [24]. Descriptions of the development and validation of the DII have been published previously [24, 26, 27]. Shivappa et al. determined inflammatory effect scores for all 45 dietary parameters (negative for anti-inflammatory and positive for pro-inflammatory) according to 1,943 studies, as well as the average global intake and standard deviations of each parameter [24]. We calculated participants’ DII scores from their mean consumption of 31 nutrients, assessed up to five times between 2009 and 2012 (Supplementary Table S2) [20]. Z-scores of each dietary parameter were first calculated to standardize intake according to global consumption, which were then converted to centered percentiles. The centered percentiles of each dietary component were multiplied by the corresponding inflammatory effect scores, and these were summed to obtain overall DII scores. Higher DII scores indicate a more pro-inflammatory diet. Participants were categorized into four groups as published previously [11] (Group 1: DII < −2, n = 4904; Group 2: DII −2 to < 0, n = 7572; Group 3: DII 0 to < 2, n = 6386; Group 4: DII ≥ 2, n = 2611) to facilitate comparison with other studies.

### Inflammation score

A composite measure of low-grade systemic inflammation (INFLA-score) was created from high-sensitivity C-reactive protein, white blood cell count, platelet count, and neutrophil-to-lymphocyte ratio assessed at baseline blood draw. Each of these four components are established markers of systemic inflammation [28]. C-reactive protein was measured using a Beckman Coulter AU5800, and a Coulter LH750 was used for white blood cells, platelets, neutrophils, and lymphocytes. Further details on the UK Biobank’s procedures for collection and processing of blood samples are available elsewhere [29]. For all four inflammatory markers, concentrations in the 7th to 10th deciles were assigned corresponding values from + 1 to + 4, and those in the 1st to 4th deciles were assigned values from − 4 to − 1 [28]. These values were summed to obtain the overall INFLA-score, ranging from −16 to + 16, with higher scores reflecting a higher level of inflammation.

### Brain MRI acquisition and pre-processing

Between 2014 and 2020, participants underwent structural and functional brain MRI at one of three imaging centers located in Manchester, Reading, and Newcastle using a Siemens Skyra 3T scanner (software platform VD13A SP4) and a standard Siemens 32-channel receive head coil. A detailed description of UK Biobank’s MRI protocol and processing pipeline has been described previously [30, 31] and is summarized in Supplementary Table S3. Briefly, 1,079 imaging-derived phenotypes (IDPs) were obtained from six modalities. These included T1-weighted MRI (n = 165), T2-FLAIR (n = 1), T2* (n = 14), diffusion MRI (n = 675), resting-state functional MRI (fMRI; n = 210), and task fMRI (n = 14). The complete list of 1,079 IDPs is presented in Supplementary Table S4. T1-weighted MRI measures the volumes of brain tissues and structures, T2-FLAIR detects white matter lesions which indicate vascular damage, T2* detects brain lesions in subcortical structures, diffusion MRI estimates microstructural integrity of white matter, resting-state fMRI assesses brain activity at rest (reflecting functional connectivity between regions), and task fMRI measures activity during a task or experience (in this case, a face/shapes matching task engaging sensory, motor, and cognitive systems).

### Estimation of brain age and brain age gap

The workflow for modeling brain age is displayed in Supplementary Fig. S1. We estimated brain age in all participants with complete MRI data (n = 34,296) based on previously established methods [32] using 1079 IDPs [32]. Participants with missing values for any of the IDPs were omitted from the analysis (Supplementary Table S4). In brain age modeling, brain age should theoretically match chronological age in people who are aging normally and free of diseases, so we created a subset of healthy participants free of ICD-10 diagnoses (stroke: n = 4056; T2D: n = 2443), self-reported long-term illness, disability, or frailty (n = 2188), and self-reported fair or poor health status (n = 2178), leaving a total of 4355 healthy participants. This healthy subset was then randomly split into a training set (n = 3484) and validation set (n = 871) using a 4:1 ratio. The testing set included all remaining participants with complete IDPs (n = 29,941).

All IDPs were Z-score standardized before training to eliminate the influence of different dimensionality. To prevent data leakage, the means and standard deviations used for this standardization were calculated exclusively from the training dataset. Nine machine learning models (eXtreme Gradient Boosting [XGBoost], Least Absolute Shrinkage and Selection Operator [LASSO] regression, and Support Vector Regression [SVR], using three versions of feature selection for each—no feature selection, FeatureWiz, and Recursive Feature Elimination with Cross Validation [RFECV]) were trained for the assessment of brain age using the standardized training dataset (Supplementary Tables S5 and S6). Bayesian optimization was performed to optimize model hyperparameters, and mean absolute error (MAE) was used to compare performance of the nine models on the standardized validation dataset. The LASSO model without feature selection achieved the lowest MAE in the validation dataset (Supplementary Table S7) and was therefore selected to predict brain age for the remaining participants with complete brain MRI data (testing subset, n = 29,941). A total of 285 IDPs were identified as significant contributors to brain age in this model (Supplementary Table S8). Brain age models tend to overpredict in younger people and underpredict in older individuals [33]. We adjusted for this bias by correcting for chronological age according to a method proposed by Cole [32] (*corrected brain age* = *[original brain age – β/α]*, where coefficients *α* and *β* are the slope and intercept of: *brain age*_*training set*_ = α*chronological age_*training set*_ + *β*) (Supplementary Fig. S2). Brain age was estimated using sklearn 1.2.0, featurewiz 0.2.3, scikit-optimize 0.9.0, and xgboost 1.7.2 in Python 3.8.0.

Brain age gap (BAG) was computed by subtracting chronological age at the time of MRI scan from corrected brain age. BAG > 0 denotes an older brain age compared to chronological age (i.e. accelerated brain aging), whereas BAG < 0 indicates a younger and healthier brain than expected.

### Assessment of covariates

#### Sociodemographic characteristics

Age, sex, race (white vs non-white), education (college degree vs not), and smoking status (never, current, or former) were self-reported at baseline. Physical activity was ascertained using the International Physical Activity Questionnaire (IPAQ) [34] and categorized as low, moderate, or high. Socioeconomic status was assessed using the Townsend deprivation index (TDI), a measure of socioeconomic deprivation based on neighborhood levels of unemployment, household overcrowding, car non-ownership, and home non-ownership [35]. Higher TDI scores indicate greater socioeconomic deprivation.

#### Genetic risk for dementia

DNA extracted from baseline blood samples was genotyped using the UK Biobank Lung Exome Variant Evaluation (UK BiLEVE) Axiom Array or Affymetrix UK Biobank Axion Array, which analyze 807,411 and 825,927 markers, respectively, across the entire genome [19, 36]. A polygenic risk score for Alzheimer’s disease (PRS_AD_) was constructed according to AD-related single nucleotide polymorphisms (SNPs) selected through meta-analysis of multiple external genome-wide association studies [36]. AD-related alleles were weighted according to their strength of association with AD. Each participant’s PRS_AD_ was ascertained by computing the Z-standardized sum of weighted AD-related alleles. The PRS_AD_ has shown high predictive performance for incident AD and all-cause dementia and is comparable to that of other well-established polygenic risk scores [36]. Participants were classified as low, moderate, or high risk tertiles. *APOE* genotype was also determined from single-nucleotide polymorphisms rs429358 and rs7412, and participants were classified as ε4 allele (*APOE*4) carriers or non-carriers.

#### Cardiometabolic variables

Body mass index (BMI; kg/m^2^) was calculated from height and weight measured at assessment, and participants were classified as underweight (< 18.5 kg/m^2^), normal (18.5 to < 25 kg/m^2^), overweight (25 to < 30 kg/m^2^), or obese (≥ 30 kg/m^2^). Waist circumference was also measured at baseline assessment, and we categorized participants as normal (< 88 cm for women or < 102 cm for men) or high risk (≥ 88 cm for women or ≥ 102 cm for men) [37]. Hypertension was identified as systolic blood pressure ≥ 140 mmHg or diastolic blood pressure ≥ 90 mmHg from baseline measurement, self-reported history of hypertension, or the use of antihypertensive medication.

Diagnoses of cardiometabolic diseases (CMDs), including cardiovascular disease (CVD; myocardial infarction, coronary artery disease, heart failure, atrial fibrillation, and angina) and type 2 diabetes (T2D), were ascertained at baseline. CVD status was obtained through self-reported medical history and medical records. Participants meeting any of the following criteria at baseline were classified as having T2D: medical record of T2D, self-reported history of T2D, use of glucose-lowering medication, hemoglobin A1c ≥ 6.5%, or fasting plasma glucose ≥ 126 mg/dL [38]. All recorded diagnoses were classified according to codes from the International Classification of Diseases (ICD) 9 and 10.

### Statistical analysis

Differences in sample characteristics by DII groups were examined using analysis of variance (ANOVA) for continuous variables and χ^2^ tests for categorical variables. Associations of DII with BAG were analyzed using linear regression. DII scores were examined continuously and as four groups (DII < −2 as reference). Models were first adjusted for age, sex, race, education, and socioeconomic status, followed by further adjustment for energy intake, BMI, smoking status, physical activity, CVD, T2D, hypertension, and PRS_AD_. Interaction and stratified analyses by age (middle-aged, 40–59 years; older-aged, ≥ 60 years), PRS_AD_, and *APOE*4 status were also performed.

The association between DII and INFLA-score was assessed using linear regression. Mediation analysis was performed using linear regression with the *mediate* function in the R package “Mediation” using bootstrapping (1,000 simulations) to determine whether INFLA-score mediated the association between continuous DII scores and BAG. This involved four separate models: (1) linear regression of BAG according to DII, (2) linear regression of INFLA-score according to DII, (3) linear regression of BAG according to both DII and INFLA-score, and (4) mediation analysis based on models from steps 2 and 3 to estimate the average causal mediation effect (ACME) of INFLA-score, the average direct effect (ADE) of DII, and the proportion mediated by INFLA-score. Mediation analysis was performed first using basic adjustment, followed by multivariable adjustment. Given that INFLA-score was determined from inflammatory markers measured at the baseline assessment visit, to minimize the risk of reverse causality, DII scores used in this analysis were computed from the same baseline assessment and did not include later dietary assessments (available for n = 4439 participants).

#### Sensitivity analysis

Analyses were repeated (1) with imputed data for missing covariates using multiple imputation by chained equations; (2) among those with only 1 (n = 6244), ≥ 2 (n = 15,229), or ≥ 4 (n = 4687) dietary assessments; (3) after excluding those who indicated atypical dietary intake on the previous day (n = 8272); and (4) including only those with ≥ 2 typical dietary assessments (n = 8491). To further verify independence of the DII-BAG association from genetic risk for dementia, we performed this analysis adjusting for *APOE*4 status in place of PRS_AD_.

#### Supplementary analysis

We also examined the interaction effects of DII with sex, BMI, waist circumference, and baseline CMD status in relation to BAG.

Analyses were performed using R Studio version 2023.06.1 + 524, © 2009–2023, Posit Software, PBC, with statistical significance reported at *p* < 0.05.

## Results

### Sample characteristics

The mean (SD) age of participants (N = 21,473) at baseline was 54.9 (7.5) years, and 11,508 (53.6%) were female. Participants underwent MRI approximately 8.7 years later (mean [SD] age at MRI = 63.7 [7.6] years), and the mean (SD) brain age was 64.0 (9.1) years. Participant characteristics by DII groups are presented in Table 1. Compared to those with an anti-inflammatory diet (group 1), those with a pro-inflammatory diet (group 4) were more likely to be younger, female, current smokers, and to have a higher BMI and waist circumference, higher INFLA-score, lower energy intake, and lower socioeconomic status. Those in group 4 were also less likely to be white, college-educated, and physical activity. Overall, this sample is similar to the UK Biobank neuroimaging cohort (Supplementary Table S9), and younger and healthier than excluded participants from the larger UK Biobank population (Supplementary Table S10).

**Table 1:** Baseline sample characteristics by DII

Characteristic	Dietary inflammatory index				
	Group 1 (< −2) N = 4904	Group 2 (−2 to < 0) N = 7572	Group 3 (0 to < 2) N = 6386	Group 4 (≥ 2) N = 2611	*p*-value
Age at baseline (y), M ± SD	55.9 ± 7.4	55.1 ± 7.4	54.5 ± 7.6	53.6 ± 7.5	< 0.001
Age at MRI (y), M ± SD	64.7 ± 7.5	63.8 ± 7.5	63.2 ± 7.7	62.4 ± 7.6	< 0.001
Sex, N (%)					< 0.001
Male	2617 (53.4)	3574 (47.2)	2716 (42.5)	1058 (40.5)	
Female	2287 (46.6)	3998 (52.8)	3670 (57.5)	1553 (59.5)	
Race, N (%)					0.001
White	4585 (93.5)	7051 (93.1)	5889 (92.2)	2385 (91.3)	
Other	311 (6.3)	494 (6.5)	484 (7.6)	216 (8.3)	
College/university education, N (%)					< 0.001
Yes	2543 (51.9)	4095 (54.1)	3091 (48.4)	1087 (41.6)	
No	2353 (48.0)	3462 (45.7)	3279 (51.3)	1516 (58.1)	
Socioeconomic status, M ± SD	−2.1 ± 2.6	−2.1 ± 2.6	−1.8 ± 2.7	−1.6 ± 2.8	< 0.001
Smoking status, N (%)					< 0.001
Never	2968 (60.5)	4713 (62.2)	3922 (61.4)	1560 (59.7)	
Former	1666 (34.0)	2488 (32.9)	2080 (32.6)	810 (31.0)	
Current	260 (5.3)	357 (4.7)	366 (5.7)	235 (9.0)	
Physical activity, N (%)					< 0.001
Low	553 (11.3)	1187 (15.7)	1129 (17.7)	509 (19.5)	
Moderate	1722 (35.1)	2866 (37.8)	2428 (38.0)	927 (35.5)	
High	2066 (42.1)	2574 (34.0)	2021 (31.6)	800 (30.6)	
Body mass index, N (%)					< 0.001
Underweight	18 (0.4)	26 (0.3)	34 (0.5)	12 (0.5)	
Normal	2075 (42.3)	3193 (42.2)	2632 (41.2)	937 (35.9)	
Overweight	2048 (41.8)	3194 (42.2)	2674 (41.9)	1136 (43.5)	
Obese	759 (15.5)	1152 (15.2)	1041 (16.3)	521 (20.0)	
Waist circumference, N (%)					< 0.001
Normal	3795 (77.4)	5855 (77.3)	4881 (76.4)	1859 (71.2)	
High risk	1107 (22.6)	1714 (22.6)	1502 (23.5)	747 (28.6)	
Hypertension, N (%)	1060 (21.6)	1553 (20.5)	1257 (19.7)	513 (19.6)	0.06
Cardiovascular disease, N (%)	201 (4.1)	278 (3.7)	205 (3.2)	87 (3.3)	0.07
Type 2 diabetes, N (%)	176 (3.6)	294 (3.9)	214 (3.4)	82 (3.1)	0.22
PRS_AD_, N (%)					0.15
Low	1564 (31.9)	2501 (33.0)	2119 (33.2)	848 (32.5)	
Moderate	1593 (32.5)	2437 (32.2)	2111 (33.1)	890 (34.1)	
High	1673 (34.1)	2497 (33.0)	2035 (31.9)	827 (31.7)	
*APOE*4 status, N (%)					0.25
No	2993 (61.0)	4657 (61.5)	3906 (61.2)	1613 (61.8)	
Yes	1202 (24.5)	1725 (22.8)	1451 (22.7)	599 (22.9)	
INFLA-score, M ± SD	−2.0 ± 5.8	−1.6 ± 5.8	−1.1 ± 5.7	−0.6 ± 5.9	< 0.001
Daily energy intake (kcal), M ± SD	2420 ± 484	2135 ± 432	1873 ± 394	1607 ± 392	< 0.001

Cardiovascular disease included myocardial infarction, coronary artery disease, heart failure, atrial fibrillation, or angina

INFLA-score, inflammation score; MRI, magnetic resonance imaging; PRS_AD_, Alzheimer’s disease polygenic risk score

*Missing data*: race = 58, education = 47, socioeconomic status = 20, smoking status = 48, physical activity = 2,691, body mass index = 21, waist circumference = 13, hypertension = 5, type 2 diabetes = 2; PRS_AD_ = 378; *APOE*4 status = 3,327, INFLA-score = 2,429

DII scores ranged from −6.22 to 5.38, which, as expected, is somewhat narrower than the theoretical lower (−8.87) and upper (7.98) limits of the DII when all 45 parameters are available [25]. A total of 15,229 (71%) participants had at least 2 dietary assessments (Supplementary Table S11). There was a modest correlation between DII scores calculated from participants’ earliest dietary assessments and scores averaged from subsequent assessments (Pearson correlation coefficient = 0.45, *p* < 0.001), indicating dietary stability between 2009 and 2012. Bland–Altman plots comparing DII scores between (1) baseline and visit 5 assessments, and (2) participants’ first and last visits suggest agreement between DII scores over time (Supplementary Fig. S3).

### The association of DII with BAG

**Fig. 2:**
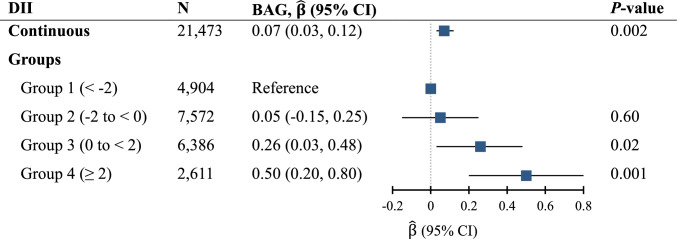
The association of DII with BAG. Models were adjusted for age, sex, race, education, socioeconomic status, energy intake, body mass index, smoking status, physical activity, cardiovascular disease, type 2 diabetes, hypertension, and Alzheimer’s disease polygenic risk score. *Abbreviations*: BAG, brain age gap; CI, confidence interval; DII, Dietary Inflammatory Index

**Fig. 3:**
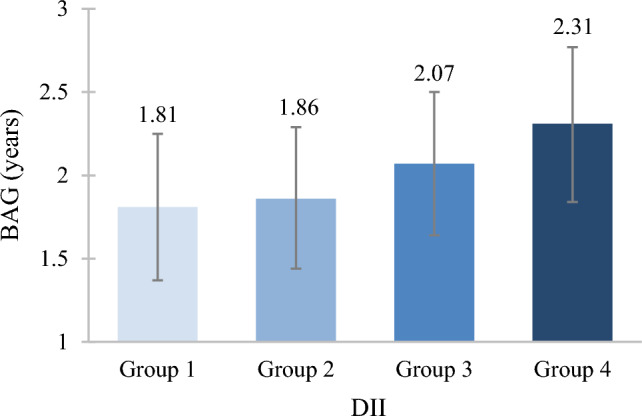
Least-squares means and 95% confidence intervals of BAG based on DII groups from multivariable-adjusted regression. *Abbreviations*: BAG, brain age gap; DII, Dietary Inflammatory Index

Each unit increase in DII was associated with significantly greater BAG by $\widehat{\beta }$ (95% confidence interval [CI]) = 0.07 (0.03, 0.12) years (Fig. 2). In analysis of DII groups, those in groups 3 and 4 had a greater BAG by 0.26 [0.03, 0.48] and 0.50 [0.20, 0.80]) years, respectively, compared to group 1. Least-squares means and 95% CIs of BAG according to DII groups from multivariable-adjusted models are shown in Fig. 3.

### Differences in the association of DII with BAG by age and genetic risk for dementia

**Fig. 4:**
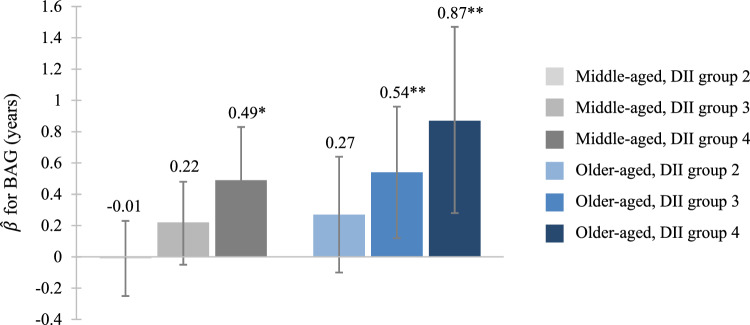
Beta-hat coefficients and 95% confidence intervals of BAG based on DII groups from age-stratified multivariable-adjusted regression. *p < 0.05 compared to middle-aged, group 1; **p < 0.05 compared to older-aged, group 1. *Abbreviations*: BAG, brain age gap; DII, Dietary Inflammatory Index

There were no significant interactions between DII and age in relation to BAG (*p*s ≥ 0.15; Supplementary Table S12). However, the DII-BAG association was pronounced in older compared to middle-aged adults when DII was analyzed both continuously (older adults: 0.13 [0.04, 0.22]; middle-aged adults: 0.08 [0.02, 0.13] and as four groups (older adults: group 3, 0.54 [0.12, 0.96]; group 4, 0.87 [0.28, 1.47]; middle-aged adults: group 3, 0.22 [−0.05, 0.48]; group 4, 0.49 [0.14, 0.83]) (Supplementary Table S13). Relationships of DII groups with BAG by age group are displayed in Fig. 4.

There were no significant interactions between DII and PRS_AD_ (*p*s ≥ 0.44) or *APOE*4 (*p*s ≥ 0.23) in relation to BAG (Supplementary Table S12). In stratified analysis, higher DII was associated with greater BAG in those with moderate PRS_AD_ when DII scores were analyzed both continuously (0.09 [0.01, 0.17]) and as groups (group 4, 0.65 [0.13, 1.18]), but this association was attenuated among those with low and high genetic risk (*p*s ≥ 0.07) (Supplementary Table S13). In *APOE*-stratified analysis, higher DII was associated with greater BAG in *APOE*4 non-carriers (continuous DII, 0.09 [0.04, 0.15]; group 4, 0.48 [0.10, 0.86]) and attenuated among *APOE*4 carriers (*p*s ≥ 0.18).

### Mediation analysis of INFLA-score in the association of DII with BAG

**Fig. 5:**
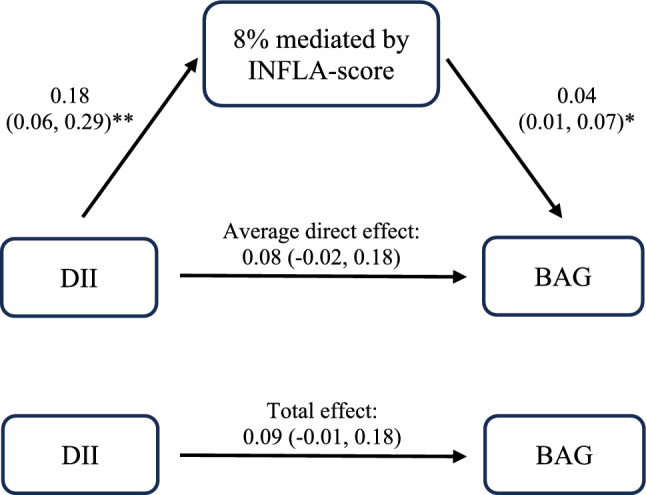
Partial mediation of the association between DII and BAG by INFLA-score from multivariable adjustment. Values are $\widehat{\beta }$ (95% confidence interval). *p < 0.05; **p < 0.01. *Abbreviations*: BAG, brain age gap; DII, Dietary Inflammatory Index; INFLA-score, inflammation score

Higher DII at baseline was associated with significantly higher INFLA-score when examined both continuously (0.18 [0.06, 0.29]) and as groups (group 4, 1.22 [0.40, 2.05]) (Supplementary Table S14). In mediation analysis, INFLA-score accounted for 8% of the association between DII and BAG ($\widehat{\beta }$ of the average causal mediation effect = 0.01 [0.001, 0.02]; Table 2 and Fig. 5).

**Table 2:** Mediation analysis of INFLA-score in the association of DII with BAG (n = 4,439)

INFLA-score	Brain Age Gap (BAG)	
	Basic-adjusted	Multi-adjusted
	$\widehat{\upbeta }$(95% CI)	$\widehat{\upbeta }$(95% CI)
ACME	0.01 (0.003, 0.02)**	0.01 (0.001, 0.02)*
ADE	0.03 (−0.05, 0.11)	0.08 (−0.02, 0.18)
Total effect	0.04 (−0.04, 0.12)	0.09 (−0.01, 0.18)
Prop. mediated	0.23 (−2.09, 1.90)	0.08 (−0.21, 0.49)

Basic adjustment included age, sex, race, education, and socioeconomic status. Multivariable adjustment included age, sex, race, education, socioeconomic status, energy intake, body mass index, smoking status, physical activity, cardiovascular disease, type 2 diabetes, hypertension, and Alzheimer’s disease polygenic risk score

^*^p < 0.05; **p < 0.01

ACME, average causal mediation effect; ADE, average direct effect; BAG, brain age gap; CI, confidence interval; INFLA-score, inflammation score; prop. mediated, proportion mediated

### Sensitivity and supplementary analyses

Associations between DII and BAG were similar following imputation of missing covariates (Supplementary Table S15) and among those with ≥ 2 dietary assessments (n = 15,229), strengthened in those reporting a typical diet (n = 13,201), and attenuated in those with only one dietary assessment (n = 6244) (Supplementary Table S16). DII-BAG associations were mostly strengthened among those with ≥ 4 dietary assessments, with the exception of group 4 which did not meet significance. This may be due to limited power, as only 368 of these 4,687 participants had DII scores ≥ 2. In those with ≥ 2 assessments of typical dietary intake (n = 8491), BAG among those in DII group 4 (0.59 [0.08, 1.11] years) was greater compared to the full sample, although the association using continuous DII was attenuated (*p* = 0.06).

The association between DII and BAG was similar after adjusting for *APOE*4 status in place of PRS_AD_ (Supplementary Table S17). In interaction analysis between DII and sex, males in DII group 3 had greater BAG compared to females (*p* for interaction = 0.03) (Supplementary Table S18). No interactions were detected between DII and BMI, waist circumference, or CMDs.

## Discussion

In this large community-based, prospective study, UK adults consuming a pro-inflammatory diet had advanced brain age by half a year. This association was almost doubled to 0.87 years in older adults, and was stronger among *APOE*4 non-carriers and those with a moderate PRS_AD_. INFLA-score had a significant mediating role in this association, indicating that diet may exacerbate brain aging in part by increasing systemic inflammation. Previous research has shown that systemic inflammation may have a detrimental impact on brain and cognitive function [39–41] and contribute to dementia pathology [2]. Our results expand on these reports by identifying diet-related inflammation as a potential modifiable factor for preserving brain integrity.

Our finding of advanced brain age among those consuming a more pro-inflammatory diet is largely consistent with investigations of individual brain measures. A recent investigation of 24,109 UK Biobank participants reported smaller volumes of hippocampal gray matter and larger volumes of white matter hyperintensities in relation to higher DII [12]. Higher DII was also associated with smaller gray matter and total brain volumes and larger lateral ventricular volumes in 1897 participants from the Framingham Heart Study Offspring cohort [14]. A study of 330 dementia-free older adults from the Washington Heights-Inwood Columbia Aging Project (WHICAP) similarly found that an inflammatory-related nutrient pattern was associated with smaller gray matter, white matter, and total brain volumes [42]. Another study of 641 middle-aged and older adults from the Cognition and Diabetes in Older Tasmanians study found little evidence of an association between DII and brain structures, although unlike ours and the previous studies discussed, it was a cross-sectional examination of diet and MRI scans collected within two weeks of each other [43]. Building upon these reports of individual brain volumes by integrating 1079 structural and functional measures, our findings indicate that a pro-inflammatory diet may have a cumulative detrimental impact on overall brain integrity.

Older adults appeared especially vulnerable to the relation between a pro-inflammatory diet and advanced brain age, with a 1.8-fold stronger association compared to middle-aged adults. This finding is consistent with the increasing risk of immune dysregulation, systemic inflammation, and neurodegeneration with age [15, 44]. Older adults may therefore be more susceptible to harmful effects of diet on both systemic inflammation and the brain compared to younger adults, and could possibly benefit more from an anti-inflammatory diet.

The association between a pro-inflammatory diet and brain age varied somewhat by genetic risk for dementia, with significant associations only among those with moderate PRS_AD_ and *APOE*4 non-carriers. DII variation within subgroups of PRS_AD_ tertiles and *APOE*4 status was reduced, with lower amounts of people in group 4, which could have led to reduced power to detect significant results. One previous study similarly found that higher DII scores were associated with smaller hippocampal volume only among *APOE*4 non-carriers [14]. This contrasts with some reports suggesting that saturated fat (pro-inflammatory) and poly-unsaturated fat (anti-inflammatory) may modify *APOE*4-related risk for cognitive decline and dementia [45, 46]. Investigations of larger samples are needed to confirm genetic differences, especially given the absence of interaction between DII, PRS_AD_, and *APOE*4 in our study.

The mediating role of INFLA-score provides evidence that diet may influence brain health in part by modifying systemic inflammation. INFLA-score has previously been associated with DII [47], as well as reduced volumes of individual cortical and subcortical brain regions [48]. Specific mechanisms linking peripheral inflammation with neurodegeneration are not completely understood, but are thought to involve numerous concurrent pathways [49]. In one such pathway, chronically elevated inflammatory markers can impair the blood–brain barrier and activate microglia and astrocytes, which in turn release reactive oxygen species and inflammatory cytokines [49, 50]. This can lead to endothelial and synaptic dysfunction, neuronal damage, and brain atrophy [49, 51]. Neuroinflammation also appears to play a major role in the pathogenesis of neurodegenerative diseases, including initiation of AD pathology [52]. Indirect mechanisms may further explain the connection between a pro-inflammatory diet and brain aging. For instance, the pro-inflammatory Western diet is adversely associated with cardiometabolic diseases [53] and gut microbiome composition [54], both of which are critically involved in dementia development [55, 56].

Our findings are in line with numerous investigations showing more favorable brain measures and lower risk of cognitive decline and dementia in relation to healthy dietary patterns, such as the Mediterranean, Dietary Approach to Stop Hypertension (DASH), and Mediterranean-DASH diet Intervention for Neurodegenerative Delay [MIND] diets [57, 58]. While the DII is not representative of a particular diet, it generally favors foods characteristic of these healthy dietary patterns. These include vegetables, fruits, whole grains, legumes, nuts, seeds, and seafood, which are high in fiber, polyphenols, vitamins, minerals, and omega-3 fatty acids [24, 57, 58]. Unlike these other healthy dietary patterns which do not directly measure dietary inflammatory potential, the DII enabled us to pinpoint the specific role of dietary inflammation in the association between diet and brain health. While our analyses are not able to confirm causality between a pro-inflammatory diet and brain aging, MRI scans were performed subsequently to dietary assessment, reducing the likelihood of reverse causality.

Several study limitations should be acknowledged. First, our findings may have limited generalizability given that the UK Biobank consists of nationwide volunteers, predominantly of white European descent, who are healthier and belong to a higher socioeconomic status than the general population [59]. Our sample is also younger and healthier than the excluded UK Biobank participants, which could have led to misestimation of the observed associations. Second, as with all observational studies, residual confounding may exist in our study, although we made efforts to minimize this by adjusting for sociodemographic and health characteristics. Third, there are several limitations related to dietary data. The use of self-reported dietary data introduces the possibility of recall bias. Additionally, while we used average dietary intake from multiple assessments between 2009 and 2012, dietary intake after 2012 was not available. Furthermore, the absence of data on 14 of the DII’s anti-inflammatory components could have led to misestimation of the inflammatory potential of participants’ diets. There are also inherent limitations to the DII in its ability to quantify dietary inflammatory potential. The DII primarily assesses individual nutrients, but diet encompasses a mixture of whole foods which contain numerous interacting nutrients and chemicals [60]. Moreover, while the DII assigns pro-inflammatory scores to all proteins and carbohydrates, inflammatory markers tend to be higher in relation to animal protein and refined grain consumption, but lower in relation to plant protein and whole grain intake [61, 62].

## Conclusion

Consumption of a pro-inflammatory diet was associated with advanced brain age in UK adults, which may be partially due to elevated systemic inflammation. These findings highlight the importance of adhering to an anti-inflammatory diet to help preserve brain integrity. The association between a pro-inflammatory diet and brain age was almost doubled in adults ≥ 60 years, underscoring the importance of directing dietary interventions toward older adults.

## Supplementary Information

Below is the link to the electronic supplementary material.

Supplementary file1 (PDF 1334 kb)

## Data Availability

Requests for access to UK Biobank data can be made here: https://www.ukbiobank.ac.uk/enable-your-research/apply-for-access.
