# Supplementary material for: The association between a pro-inflammatory diet and brain age in middle-aged and older adults
Source: Eur J Epidemiol. 2025 Oct 27;41(1):39–50. doi: 10.1007/s10654-025-01318-6 (PMC12881069; doi:10.1007/s10654-025-01318-6)
Supplement: Supplementary file 1 — Supplementary file1 (PDF 1334 kb) [file 10654_2025_1318_MOESM1_ESM.pdf]

Supplementary Material

The association between a pro-inflammatory diet and brain age in middle-aged and older adults

Michelle M. Dunk, Huijie Huang, Jiao Wang, Abigail Dove, Sakura Sakakibara, Jie Guo, Adrián Carballo-Casla, David A. Bennett, Weili Xu

European Journal of Epidemiology

Supplementary Table S1 Neurological disorders excluded from the study sample.

Supplementary Table S2 Components of the Dietary Inflammatory Index (DII).

Supplementary Table S3 UK Biobank brain MRI acquisition protocols.

Supplementary Table S4 Missing numbers for imaging-derived phenotypes.

Supplementary Fig. S1 Workflow used to model brain age and BAG.

Supplementary Table S5 Hyperparameter spaces of the nine candidate machine learning models for brain age estimation in Bayesian optimization.

Supplementary Table S6 Chosen hyperparameters of the nine candidate machine learning models for brain age estimation.

Supplementary Table S7 Performance comparison for nine candidate machine learning models for brain age estimation.

Supplementary Table S8 Coefficients for 285 imaging-derived phenotypes that significantly contributed to brain age estimation in LASSO regression without feature selection.

Supplementary Fig. S2 Original and corrected brain age as a function of chronological age in the (A) testing set and (B) validation set.

Supplementary Table S9 Baseline characteristics of the entire UK Biobank, UK Biobank neuroimaging cohort, and current study sample.

Supplementary Table S10 Comparison of baseline characteristics between the study sample and excluded participants.

Supplementary Table S11 Number of available dietary assessments.

Supplementary Fig. S3 Bland-Altman plots comparing Dietary Inflammatory Index (DII) scores (A) from baseline and visit 5 assessments, and (B) from participants' first and last assessments.

Supplementary Table S12 Interactions of DII with age, PRS_{AD}, and *APOE*4 in relation to BAG.

Supplementary Table S13 The association of DII with BAG stratified by age, PRS_{AD}, and *APOE*4 status.

Supplementary Table S14 Associations between baseline DII and INFLA-score.

Supplementary Table S15 The association of DII with BAG using imputed data for missing covariates.

Supplementary Table S16 The association of DII with BAG according to number and quality of dietary assessments.

Supplementary Table S17 The association of DII with BAG adjusted for *APOE*4 status.

Supplementary Table S18 Interactions of DII with sex, body mass index, waist circumference, and cardiometabolic diseases in relation to BAG.

References

Supplementary Table S1 Neurological disorders excluded from the study sample.

Self-reported illness	Code (Field ID 20001 and 20002)
Dementia or Alzheimer’s disease	1263
Parkinson’s disease	1262
Chronic degenerative neurological	1258
Guillain-Barré syndrome	1256
Multiple Sclerosis	1261
Other demyelinating diseases	1397
Stroke or ischemic stroke	1081
Brain cancer	1032
Brain hemorrhage	1491
Brain/intracranial abscess	1245
Cerebral aneurysm	1425
Cerebral palsy	1433
Encephalitis	1246
Epilepsy	1264
Head injury	1266
Infections of the nervous system	1244
Ischemic stroke	1583
Meningeal cancer	1031
Meningioma (benign)	1659
Meningitis	1247
Motor Neuron Disease	1259
Neurological injury/trauma	1240
Spina bifida	1524
Subdural hematoma	1083
Subarachnoid hemorrhage	1086
Transient ischemic attack	1082
Neuroma (benign)	1683
Other neurological problem	1434

Supplementary Table S2 Components of the Dietary Inflammatory Index (DII).

Food parameter (unit of measurement)	Food parameter-specific inflammatory effect score[a]
Alcohol* (g)	-0.278
Anthocyanidins (mg)	-0.131
β-carotene* (μg)	-0.584
Caffeine (g)	-0.110
Carbohydrate* (g)	0.097
Cholesterol* (mg)	0.110
Energy* (kcal)	0.180
Eugenol (mg)	-0.140
Total fat* (g)	0.298
Fiber* (g)	-0.663
Flavan-3-ol (mg)	-0.415
Flavones (mg)	-0.616
Flavonols (mg)	-0.467
Flavonones (mg)	-0.250
Folic acid* (μg)	-0.190
Garlic* (g)	-0.412
Ginger (g)	-0.453
Green/black tea* (g)	-0.536
Iron* (mg)	0.032
Isoflavones (mg)	-0.593
Magnesium* (mg)	-0.484
Monounsaturated fatty acids* (g)	-0.009
Niacin* (mg)	-0.246
Omega-3 fatty acids* (g)	-0.436
Omega-6 fatty acids* (g)	-0.159
Onion* (g)	-0.301
Pepper (g)	-0.131
Polyunsaturated fatty acids* (g)	-0.337
Protein* (g)	0.021
Riboflavin* (mg)	-0.068
Rosemary (mg)	-0.013
Saffron (g)	-0.140
Saturated fatty acids* (g)	0.373
Selenium* (μg)	-0.191
Thiamin* (mg)	-0.098
Thyme/oregano (mg)	-0.102
Trans fatty acids* (g)	0.229
Turmeric (mg)	-0.785
Vitamin A* (RE)	-0.401
Vitamin B6* (mg)	-0.365
Vitamin B12* (μg)	0.106
Vitamin C* (mg)	-0.424
Vitamin D* (μg)	-0.446
Vitamin E* (mg)	-0.419
Zinc* (mg)	-0.313

[a] Food parameter-specific inflammatory effect scores were used to calculate Dietary Inflammatory Index scores [1]. A negative value indicates an anti-inflammatory effect, and a positive value indicates a pro-inflammatory effect.

*Available for calculation of DII scores in the UK Biobank.

Abbreviations: RE, retinol equivalents.

Supplementary Table S3 UK Biobank brain MRI acquisition protocols.

**Modality**	**Duration (minutes)**	**Voxel, Matrix**	**Key Parameters**
T1	4:54	1 × 1 × 1 mm<br>208 × 256 × 256	3D MPRAGE, sagittal, R = 2, TI/TR = 880/2000 ms
T2 FLAIR	5:52	1.05 × 1.0 × 1.0 mm<br>192 × 256 × 256	FLAIR, 3D SPACE, sagittal, R = 2, PF 7/8, fat sat, TI/TR = 1800/5000 ms, elliptical
T2*	2:34	0.8 × 0.8 × 3 mm<br>256 × 288 × 48	3D GRE, axial, R = 2, PF 7/8 TE1/TE2/TR = 9.4/20/27 ms
Diffusion MRI	7:08	2.0 × 2.0 × 2.0 mm<br>104 × 104 × 72	MB = 3, R = 1, TE/TR = 92/3600 ms, PF 6/8, fat sat, b = 0 s/mm^2 (5x + 3 × phase-encoding reversed), b = 1000 s/mm^2 (50×), b = 2000 s/mm^2 (50×)
Resting-state fMRI	6:10	2.4 × 2.4 × 2.4 mm<br>88 × 88 × 64	TE/TR = 39/735 ms, MB = 8, R = 1, flip angle 52°, fat sat
Task fMRI	4:13	2.4 × 2.4 × 2.4 mm<br>88 × 88 × 64	Acquisition same as resting-state fMRI. Task is faces/shapes “emotion” task.

All non-EPI scans were pre-scan normalized (on-scanner bias-field corrected). Gradient distortion correction was deselected on the scanner and applied in post-processing. Table modified from Alfaro-Almagro et al. [2].

Abbreviations: R, in-plane acceleration factor; MB, multiband factor; PF, partial Fourier.

Supplementary Table S4 Missing numbers for imaging-derived phenotypes.

Phenotype	Field ID	Modality	Missing
Volumetric scaling from T1 head image to standard space	25000	T1-weighted MRI	3
Volume of peripheral cortical grey matter (normalized for head size)	25001	T1-weighted MRI	3
Volume of peripheral cortical grey matter	25002	T1-weighted MRI	3
Volume of ventricular cerebrospinal fluid (normalized for head size)	25003	T1-weighted MRI	3
Volume of ventricular cerebrospinal fluid	25004	T1-weighted MRI	3
Volume of grey matter (normalized for head size)	25005	T1-weighted MRI	3
Volume of grey matter	25006	T1-weighted MRI	3
Volume of white matter (normalized for head size)	25007	T1-weighted MRI	3
Volume of white matter	25008	T1-weighted MRI	3
Volume of brain, grey+white matter (normalized for head size)	25009	T1-weighted MRI	3
Volume of brain, grey+white matter	25010	T1-weighted MRI	3
Volume of thalamus (left)	25011	T1-weighted MRI	20
Volume of thalamus (right)	25012	T1-weighted MRI	20
Volume of caudate (left)	25013	T1-weighted MRI	20
Volume of caudate (right)	25014	T1-weighted MRI	20
Volume of putamen (left)	25015	T1-weighted MRI	20
Volume of putamen (right)	25016	T1-weighted MRI	20
Volume of pallidum (left)	25017	T1-weighted MRI	20
Volume of pallidum (right)	25018	T1-weighted MRI	20
Volume of hippocampus (left)	25019	T1-weighted MRI	20
Volume of hippocampus (right)	25020	T1-weighted MRI	20
Volume of amygdala (left)	25021	T1-weighted MRI	20
Volume of amygdala (right)	25022	T1-weighted MRI	20
Volume of accumbens (left)	25023	T1-weighted MRI	20
Volume of accumbens (right)	25024	T1-weighted MRI	20
Volume of brain stem + 4th ventricle	25025	T1-weighted MRI	20
Volume of grey matter in Frontal Pole (left)	25782	T1-weighted MRI	8
Volume of grey matter in Frontal Pole (right)	25783	T1-weighted MRI	8
Volume of grey matter in Insular Cortex (left)	25784	T1-weighted MRI	8
Volume of grey matter in Insular Cortex (right)	25785	T1-weighted MRI	8
Volume of grey matter in Superior Frontal Gyrus (left)	25786	T1-weighted MRI	8
Volume of grey matter in Superior Frontal Gyrus (right)	25787	T1-weighted MRI	8
Volume of grey matter in Middle Frontal Gyrus (left)	25788	T1-weighted MRI	8
Volume of grey matter in Middle Frontal Gyrus (right)	25789	T1-weighted MRI	8
Volume of grey matter in Inferior Frontal Gyrus, pars triangularis (left)	25790	T1-weighted MRI	8
Volume of grey matter in Inferior Frontal Gyrus, pars triangularis (right)	25791	T1-weighted MRI	8
Volume of grey matter in Inferior Frontal Gyrus, pars opercularis (left)	25792	T1-weighted MRI	8
Volume of grey matter in Inferior Frontal Gyrus, pars opercularis (right)	25793	T1-weighted MRI	8

Volume of grey matter in Precentral Gyrus (left)	25794	T1-weighted MRI	8
Volume of grey matter in Precentral Gyrus (right)	25795	T1-weighted MRI	8
Volume of grey matter in Temporal Pole (left)	25796	T1-weighted MRI	8
Volume of grey matter in Temporal Pole (right)	25797	T1-weighted MRI	8
Volume of grey matter in Superior Temporal Gyrus, anterior division (left)	25798	T1-weighted MRI	8
Volume of grey matter in Superior Temporal Gyrus, anterior division (right)	25799	T1-weighted MRI	8
Volume of grey matter in Superior Temporal Gyrus, posterior division (left)	25800	T1-weighted MRI	8
Volume of grey matter in Superior Temporal Gyrus, posterior division (right)	25801	T1-weighted MRI	8
Volume of grey matter in Middle Temporal Gyrus, anterior division (left)	25802	T1-weighted MRI	8
Volume of grey matter in Middle Temporal Gyrus, anterior division (right)	25803	T1-weighted MRI	8
Volume of grey matter in Middle Temporal Gyrus, posterior division (left)	25804	T1-weighted MRI	8
Volume of grey matter in Middle Temporal Gyrus, posterior division (right)	25805	T1-weighted MRI	8
Volume of grey matter in Middle Temporal Gyrus, temporooccipital part (left)	25806	T1-weighted MRI	8
Volume of grey matter in Middle Temporal Gyrus, temporooccipital part (right)	25807	T1-weighted MRI	8
Volume of grey matter in Inferior Temporal Gyrus, anterior division (left)	25808	T1-weighted MRI	8
Volume of grey matter in Inferior Temporal Gyrus, anterior division (right)	25809	T1-weighted MRI	8
Volume of grey matter in Inferior Temporal Gyrus, posterior division (left)	25810	T1-weighted MRI	8
Volume of grey matter in Inferior Temporal Gyrus, posterior division (right)	25811	T1-weighted MRI	8
Volume of grey matter in Inferior Temporal Gyrus, temporooccipital part (left)	25812	T1-weighted MRI	8
Volume of grey matter in Inferior Temporal Gyrus, temporooccipital part (right)	25813	T1-weighted MRI	8
Volume of grey matter in Postcentral Gyrus (left)	25814	T1-weighted MRI	8
Volume of grey matter in Postcentral Gyrus (right)	25815	T1-weighted MRI	8
Volume of grey matter in Superior Parietal Lobule (left)	25816	T1-weighted MRI	8
Volume of grey matter in Superior Parietal Lobule (right)	25817	T1-weighted MRI	8
Volume of grey matter in Supramarginal Gyrus, anterior division (left)	25818	T1-weighted MRI	8
Volume of grey matter in Supramarginal Gyrus, anterior division (right)	25819	T1-weighted MRI	8
Volume of grey matter in Supramarginal Gyrus, posterior division (left)	25820	T1-weighted MRI	8
Volume of grey matter in Supramarginal Gyrus, posterior division (right)	25821	T1-weighted MRI	8
Volume of grey matter in Angular Gyrus (left)	25822	T1-weighted MRI	8
Volume of grey matter in Angular Gyrus (right)	25823	T1-weighted MRI	8
Volume of grey matter in Lateral Occipital Cortex, superior division (left)	25824	T1-weighted MRI	8
Volume of grey matter in Lateral Occipital Cortex, superior division (right)	25825	T1-weighted MRI	8
Volume of grey matter in Lateral Occipital Cortex, inferior division (left)	25826	T1-weighted MRI	8

Volume of grey matter in Lateral Occipital Cortex, inferior division (right)	25827	T1-weighted MRI	8
Volume of grey matter in Intracalcarine Cortex (left)	25828	T1-weighted MRI	8
Volume of grey matter in Intracalcarine Cortex (right)	25829	T1-weighted MRI	8
Volume of grey matter in Frontal Medial Cortex (left)	25830	T1-weighted MRI	8
Volume of grey matter in Frontal Medial Cortex (right)	25831	T1-weighted MRI	8
Volume of grey matter in Juxtapositional Lobule Cortex (formerly Supplementary Motor Cortex) (left)	25832	T1-weighted MRI	8
Volume of grey matter in Juxtapositional Lobule Cortex (formerly Supplementary Motor Cortex) (right)	25833	T1-weighted MRI	8
Volume of grey matter in Subcallosal Cortex (left)	25834	T1-weighted MRI	8
Volume of grey matter in Subcallosal Cortex (right)	25835	T1-weighted MRI	8
Volume of grey matter in Paracingulate Gyrus (left)	25836	T1-weighted MRI	8
Volume of grey matter in Paracingulate Gyrus (right)	25837	T1-weighted MRI	8
Volume of grey matter in Cingulate Gyrus, anterior division (left)	25838	T1-weighted MRI	8
Volume of grey matter in Cingulate Gyrus, anterior division (right)	25839	T1-weighted MRI	8
Volume of grey matter in Cingulate Gyrus, posterior division (left)	25840	T1-weighted MRI	8
Volume of grey matter in Cingulate Gyrus, posterior division (right)	25841	T1-weighted MRI	8
Volume of grey matter in Precuneous Cortex (left)	25842	T1-weighted MRI	8
Volume of grey matter in Precuneous Cortex (right)	25843	T1-weighted MRI	8
Volume of grey matter in Cuneal Cortex (left)	25844	T1-weighted MRI	8
Volume of grey matter in Cuneal Cortex (right)	25845	T1-weighted MRI	8
Volume of grey matter in Frontal Orbital Cortex (left)	25846	T1-weighted MRI	8
Volume of grey matter in Frontal Orbital Cortex (right)	25847	T1-weighted MRI	8
Volume of grey matter in Parahippocampal Gyrus, anterior division (left)	25848	T1-weighted MRI	8
Volume of grey matter in Parahippocampal Gyrus, anterior division (right)	25849	T1-weighted MRI	8
Volume of grey matter in Parahippocampal Gyrus, posterior division (left)	25850	T1-weighted MRI	8
Volume of grey matter in Parahippocampal Gyrus, posterior division (right)	25851	T1-weighted MRI	8
Volume of grey matter in Lingual Gyrus (left)	25852	T1-weighted MRI	8
Volume of grey matter in Lingual Gyrus (right)	25853	T1-weighted MRI	8
Volume of grey matter in Temporal Fusiform Cortex, anterior division (left)	25854	T1-weighted MRI	8
Volume of grey matter in Temporal Fusiform Cortex, anterior division (right)	25855	T1-weighted MRI	8
Volume of grey matter in Temporal Fusiform Cortex, posterior division (left)	25856	T1-weighted MRI	8
Volume of grey matter in Temporal Fusiform Cortex, posterior division (right)	25857	T1-weighted MRI	8
Volume of grey matter in Temporal Occipital Fusiform Cortex (left)	25858	T1-weighted MRI	8
Volume of grey matter in Temporal Occipital Fusiform Cortex (right)	25859	T1-weighted MRI	8
Volume of grey matter in Occipital Fusiform Gyrus (left)	25860	T1-weighted MRI	8
Volume of grey matter in Occipital Fusiform Gyrus (right)	25861	T1-weighted MRI	8
Volume of grey matter in Frontal Operculum Cortex (left)	25862	T1-weighted MRI	8

Volume of grey matter in Frontal Operculum Cortex (right)	25863	T1-weighted MRI	8
Volume of grey matter in Central Opercular Cortex (left)	25864	T1-weighted MRI	8
Volume of grey matter in Central Opercular Cortex (right)	25865	T1-weighted MRI	8
Volume of grey matter in Parietal Operculum Cortex (left)	25866	T1-weighted MRI	8
Volume of grey matter in Parietal Operculum Cortex (right)	25867	T1-weighted MRI	8
Volume of grey matter in Planum Polare (left)	25868	T1-weighted MRI	8
Volume of grey matter in Planum Polare (right)	25869	T1-weighted MRI	8
Volume of grey matter in Heschl's Gyrus (includes H1 and H2) (left)	25870	T1-weighted MRI	8
Volume of grey matter in Heschl's Gyrus (includes H1 and H2) (right)	25871	T1-weighted MRI	8
Volume of grey matter in Planum Temporale (left)	25872	T1-weighted MRI	8
Volume of grey matter in Planum Temporale (right)	25873	T1-weighted MRI	8
Volume of grey matter in Supracalcarine Cortex (left)	25874	T1-weighted MRI	8
Volume of grey matter in Supracalcarine Cortex (right)	25875	T1-weighted MRI	8
Volume of grey matter in Occipital Pole (left)	25876	T1-weighted MRI	8
Volume of grey matter in Occipital Pole (right)	25877	T1-weighted MRI	8
Volume of grey matter in Thalamus (left)	25878	T1-weighted MRI	8
Volume of grey matter in Thalamus (right)	25879	T1-weighted MRI	8
Volume of grey matter in Caudate (left)	25880	T1-weighted MRI	8
Volume of grey matter in Caudate (right)	25881	T1-weighted MRI	8
Volume of grey matter in Putamen (left)	25882	T1-weighted MRI	8
Volume of grey matter in Putamen (right)	25883	T1-weighted MRI	8
Volume of grey matter in Pallidum (left)	25884	T1-weighted MRI	8
Volume of grey matter in Pallidum (right)	25885	T1-weighted MRI	8
Volume of grey matter in Hippocampus (left)	25886	T1-weighted MRI	8
Volume of grey matter in Hippocampus (right)	25887	T1-weighted MRI	8
Volume of grey matter in Amygdala (left)	25888	T1-weighted MRI	8
Volume of grey matter in Amygdala (right)	25889	T1-weighted MRI	8
Volume of grey matter in Ventral Striatum (left)	25890	T1-weighted MRI	8
Volume of grey matter in Ventral Striatum (right)	25891	T1-weighted MRI	8
Volume of grey matter in Brain-Stem	25892	T1-weighted MRI	8
Volume of grey matter in I-IV Cerebellum (left)	25893	T1-weighted MRI	8
Volume of grey matter in I-IV Cerebellum (right)	25894	T1-weighted MRI	8
Volume of grey matter in V Cerebellum (left)	25895	T1-weighted MRI	8
Volume of grey matter in V Cerebellum (right)	25896	T1-weighted MRI	8
Volume of grey matter in VI Cerebellum (left)	25897	T1-weighted MRI	8
Volume of grey matter in VI Cerebellum (vermis)	25898	T1-weighted MRI	8
Volume of grey matter in VI Cerebellum (right)	25899	T1-weighted MRI	8
Volume of grey matter in Crus I Cerebellum (left)	25900	T1-weighted MRI	8
Volume of grey matter in Crus I Cerebellum (vermis)	25901	T1-weighted MRI	8
Volume of grey matter in Crus I Cerebellum (right)	25902	T1-weighted MRI	8
Volume of grey matter in Crus II Cerebellum (left)	25903	T1-weighted MRI	8
Volume of grey matter in Crus II Cerebellum (vermis)	25904	T1-weighted MRI	8
Volume of grey matter in Crus II Cerebellum (right)	25905	T1-weighted MRI	8

Volume of grey matter in VIIb Cerebellum (left)	25906	T1-weighted MRI	8
Volume of grey matter in VIIb Cerebellum (vermis)	25907	T1-weighted MRI	8
Volume of grey matter in VIIb Cerebellum (right)	25908	T1-weighted MRI	8
Volume of grey matter in VIIIa Cerebellum (left)	25909	T1-weighted MRI	8
Volume of grey matter in VIIIa Cerebellum (vermis)	25910	T1-weighted MRI	8
Volume of grey matter in VIIIa Cerebellum (right)	25911	T1-weighted MRI	8
Volume of grey matter in VIIIb Cerebellum (left)	25912	T1-weighted MRI	8
Volume of grey matter in VIIIb Cerebellum (vermis)	25913	T1-weighted MRI	8
Volume of grey matter in VIIIb Cerebellum (right)	25914	T1-weighted MRI	8
Volume of grey matter in IX Cerebellum (left)	25915	T1-weighted MRI	8
Volume of grey matter in IX Cerebellum (vermis)	25916	T1-weighted MRI	8
Volume of grey matter in IX Cerebellum (right)	25917	T1-weighted MRI	8
Volume of grey matter in X Cerebellum (left)	25918	T1-weighted MRI	8
Volume of grey matter in X Cerebellum (vermis)	25919	T1-weighted MRI	8
Volume of grey matter in X Cerebellum (right)	25920	T1-weighted MRI	8
Total volume of white matter hyperintensities (from T1 and T2_FLAIR images)	25781	T2-FLAIR	1372
Median T2star in thalamus (left)	25026	T2*	4065
Median T2star in thalamus (right)	25027	T2*	4065
Median T2star in caudate (left)	25028	T2*	4065
Median T2star in caudate (right)	25029	T2*	4065
Median T2star in putamen (left)	25030	T2*	4065
Median T2star in putamen (right)	25031	T2*	4065
Median T2star in pallidum (left)	25032	T2*	4065
Median T2star in pallidum (right)	25033	T2*	4065
Median T2star in hippocampus (left)	25034	T2*	4065
Median T2star in hippocampus (right)	25035	T2*	4065
Median T2star in amygdala (left)	25036	T2*	4065
Median T2star in amygdala (right)	25037	T2*	4065
Median T2star in accumbens (left)	25038	T2*	4065
Median T2star in accumbens (right)	25039	T2*	4065
Mean FA in middle cerebellar peduncle on FA skeleton	25056	Diffusion MRI	2289
Mean FA in pontine crossing tract on FA skeleton	25057	Diffusion MRI	2289
Mean FA in genu of corpus callosum on FA skeleton	25058	Diffusion MRI	2289
Mean FA in body of corpus callosum on FA skeleton	25059	Diffusion MRI	2289
Mean FA in splenium of corpus callosum on FA skeleton	25060	Diffusion MRI	2289
Mean FA in fornix on FA skeleton	25061	Diffusion MRI	2289
Mean FA in corticospinal tract on FA skeleton (right)	25062	Diffusion MRI	2289
Mean FA in corticospinal tract on FA skeleton (left)	25063	Diffusion MRI	2289
Mean FA in medial lemniscus on FA skeleton (right)	25064	Diffusion MRI	2289
Mean FA in medial lemniscus on FA skeleton (left)	25065	Diffusion MRI	2289
Mean FA in inferior cerebellar peduncle on FA skeleton (right)	25066	Diffusion MRI	2289
Mean FA in inferior cerebellar peduncle on FA skeleton (left)	25067	Diffusion MRI	2289

Mean FA in superior cerebellar peduncle on FA skeleton (right)	25068	Diffusion MRI	2289
Mean FA in superior cerebellar peduncle on FA skeleton (left)	25069	Diffusion MRI	2289
Mean FA in cerebral peduncle on FA skeleton (right)	25070	Diffusion MRI	2289
Mean FA in cerebral peduncle on FA skeleton (left)	25071	Diffusion MRI	2289
Mean FA in anterior limb of internal capsule on FA skeleton (right)	25072	Diffusion MRI	2289
Mean FA in anterior limb of internal capsule on FA skeleton (left)	25073	Diffusion MRI	2289
Mean FA in posterior limb of internal capsule on FA skeleton (right)	25074	Diffusion MRI	2289
Mean FA in posterior limb of internal capsule on FA skeleton (left)	25075	Diffusion MRI	2289
Mean FA in retrolenticular part of internal capsule on FA skeleton (right)	25076	Diffusion MRI	2289
Mean FA in retrolenticular part of internal capsule on FA skeleton (left)	25077	Diffusion MRI	2289
Mean FA in anterior corona radiata on FA skeleton (right)	25078	Diffusion MRI	2289
Mean FA in anterior corona radiata on FA skeleton (left)	25079	Diffusion MRI	2289
Mean FA in superior corona radiata on FA skeleton (right)	25080	Diffusion MRI	2289
Mean FA in superior corona radiata on FA skeleton (left)	25081	Diffusion MRI	2289
Mean FA in posterior corona radiata on FA skeleton (right)	25082	Diffusion MRI	2289
Mean FA in posterior corona radiata on FA skeleton (left)	25083	Diffusion MRI	2289
Mean FA in posterior thalamic radiation on FA skeleton (right)	25084	Diffusion MRI	2289
Mean FA in posterior thalamic radiation on FA skeleton (left)	25085	Diffusion MRI	2289
Mean FA in sagittal stratum on FA skeleton (right)	25086	Diffusion MRI	2289
Mean FA in sagittal stratum on FA skeleton (left)	25087	Diffusion MRI	2289
Mean FA in external capsule on FA skeleton (right)	25088	Diffusion MRI	2289
Mean FA in external capsule on FA skeleton (left)	25089	Diffusion MRI	2289
Mean FA in cingulum cingulate gyrus on FA skeleton (right)	25090	Diffusion MRI	2289
Mean FA in cingulum cingulate gyrus on FA skeleton (left)	25091	Diffusion MRI	2289
Mean FA in cingulum hippocampus on FA skeleton (right)	25092	Diffusion MRI	2289
Mean FA in cingulum hippocampus on FA skeleton (left)	25093	Diffusion MRI	2289
Mean FA in fornix cres+stria terminalis on FA skeleton (right)	25094	Diffusion MRI	2289
Mean FA in fornix cres+stria terminalis on FA skeleton (left)	25095	Diffusion MRI	2289
Mean FA in superior longitudinal fasciculus on FA skeleton (right)	25096	Diffusion MRI	2289
Mean FA in superior longitudinal fasciculus on FA skeleton (left)	25097	Diffusion MRI	2289
Mean FA in superior fronto-occipital fasciculus on FA skeleton (right)	25098	Diffusion MRI	2289
Mean FA in superior fronto-occipital fasciculus on FA skeleton (left)	25099	Diffusion MRI	2289
Mean FA in uncinate fasciculus on FA skeleton (right)	25100	Diffusion MRI	2289
Mean FA in uncinate fasciculus on FA skeleton (left)	25101	Diffusion MRI	2289
Mean FA in tapetum on FA skeleton (right)	25102	Diffusion MRI	2289
Mean FA in tapetum on FA skeleton (left)	25103	Diffusion MRI	2289
Mean MD in middle cerebellar peduncle on FA skeleton	25104	Diffusion MRI	2289
Mean MD in pontine crossing tract on FA skeleton	25105	Diffusion MRI	2289

Mean MD in genu of corpus callosum on FA skeleton	25106	Diffusion MRI	2289
Mean MD in body of corpus callosum on FA skeleton	25107	Diffusion MRI	2289
Mean MD in splenium of corpus callosum on FA skeleton	25108	Diffusion MRI	2289
Mean MD in fornix on FA skeleton	25109	Diffusion MRI	2289
Mean MD in corticospinal tract on FA skeleton (right)	25110	Diffusion MRI	2289
Mean MD in corticospinal tract on FA skeleton (left)	25111	Diffusion MRI	2289
Mean MD in medial lemniscus on FA skeleton (right)	25112	Diffusion MRI	2289
Mean MD in medial lemniscus on FA skeleton (left)	25113	Diffusion MRI	2289
Mean MD in inferior cerebellar peduncle on FA skeleton (right)	25114	Diffusion MRI	2289
Mean MD in inferior cerebellar peduncle on FA skeleton (left)	25115	Diffusion MRI	2289
Mean MD in superior cerebellar peduncle on FA skeleton (right)	25116	Diffusion MRI	2289
Mean MD in superior cerebellar peduncle on FA skeleton (left)	25117	Diffusion MRI	2289
Mean MD in cerebral peduncle on FA skeleton (right)	25118	Diffusion MRI	2289
Mean MD in cerebral peduncle on FA skeleton (left)	25119	Diffusion MRI	2289
Mean MD in anterior limb of internal capsule on FA skeleton (right)	25120	Diffusion MRI	2289
Mean MD in anterior limb of internal capsule on FA skeleton (left)	25121	Diffusion MRI	2289
Mean MD in posterior limb of internal capsule on FA skeleton (right)	25122	Diffusion MRI	2289
Mean MD in posterior limb of internal capsule on FA skeleton (left)	25123	Diffusion MRI	2289
Mean MD in retrolenticular part of internal capsule on FA skeleton (right)	25124	Diffusion MRI	2289
Mean MD in retrolenticular part of internal capsule on FA skeleton (left)	25125	Diffusion MRI	2289
Mean MD in anterior corona radiata on FA skeleton (right)	25126	Diffusion MRI	2289
Mean MD in anterior corona radiata on FA skeleton (left)	25127	Diffusion MRI	2289
Mean MD in superior corona radiata on FA skeleton (right)	25128	Diffusion MRI	2289
Mean MD in superior corona radiata on FA skeleton (left)	25129	Diffusion MRI	2289
Mean MD in posterior corona radiata on FA skeleton (right)	25130	Diffusion MRI	2289
Mean MD in posterior corona radiata on FA skeleton (left)	25131	Diffusion MRI	2289
Mean MD in posterior thalamic radiation on FA skeleton (right)	25132	Diffusion MRI	2289
Mean MD in posterior thalamic radiation on FA skeleton (left)	25133	Diffusion MRI	2289
Mean MD in sagittal stratum on FA skeleton (right)	25134	Diffusion MRI	2289
Mean MD in sagittal stratum on FA skeleton (left)	25135	Diffusion MRI	2289
Mean MD in external capsule on FA skeleton (right)	25136	Diffusion MRI	2289
Mean MD in external capsule on FA skeleton (left)	25137	Diffusion MRI	2289
Mean MD in cingulum cingulate gyrus on FA skeleton (right)	25138	Diffusion MRI	2289
Mean MD in cingulum cingulate gyrus on FA skeleton (left)	25139	Diffusion MRI	2289
Mean MD in cingulum hippocampus on FA skeleton (right)	25140	Diffusion MRI	2289
Mean MD in cingulum hippocampus on FA skeleton (left)	25141	Diffusion MRI	2289
Mean MD in fornix cres+stria terminalis on FA skeleton (right)	25142	Diffusion MRI	2289
Mean MD in fornix cres+stria terminalis on FA skeleton (left)	25143	Diffusion MRI	2289

Mean MD in superior longitudinal fasciculus on FA skeleton (right)	25144	Diffusion MRI	2289
Mean MD in superior longitudinal fasciculus on FA skeleton (left)	25145	Diffusion MRI	2289
Mean MD in superior fronto-occipital fasciculus on FA skeleton (right)	25146	Diffusion MRI	2289
Mean MD in superior fronto-occipital fasciculus on FA skeleton (left)	25147	Diffusion MRI	2289
Mean MD in uncinate fasciculus on FA skeleton (right)	25148	Diffusion MRI	2289
Mean MD in uncinate fasciculus on FA skeleton (left)	25149	Diffusion MRI	2289
Mean MD in tapetum on FA skeleton (right)	25150	Diffusion MRI	2289
Mean MD in tapetum on FA skeleton (left)	25151	Diffusion MRI	2289
Mean MO in middle cerebellar peduncle on FA skeleton	25152	Diffusion MRI	2289
Mean MO in pontine crossing tract on FA skeleton	25153	Diffusion MRI	2289
Mean MO in genu of corpus callosum on FA skeleton	25154	Diffusion MRI	2289
Mean MO in body of corpus callosum on FA skeleton	25155	Diffusion MRI	2289
Mean MO in splenium of corpus callosum on FA skeleton	25156	Diffusion MRI	2289
Mean MO in fornix on FA skeleton	25157	Diffusion MRI	2289
Mean MO in corticospinal tract on FA skeleton (right)	25158	Diffusion MRI	2289
Mean MO in corticospinal tract on FA skeleton (left)	25159	Diffusion MRI	2289
Mean MO in medial lemniscus on FA skeleton (right)	25160	Diffusion MRI	2289
Mean MO in medial lemniscus on FA skeleton (left)	25161	Diffusion MRI	2289
Mean MO in inferior cerebellar peduncle on FA skeleton (right)	25162	Diffusion MRI	2289
Mean MO in inferior cerebellar peduncle on FA skeleton (left)	25163	Diffusion MRI	2289
Mean MO in superior cerebellar peduncle on FA skeleton (right)	25164	Diffusion MRI	2289
Mean MO in superior cerebellar peduncle on FA skeleton (left)	25165	Diffusion MRI	2289
Mean MO in cerebral peduncle on FA skeleton (right)	25166	Diffusion MRI	2289
Mean MO in cerebral peduncle on FA skeleton (left)	25167	Diffusion MRI	2289
Mean MO in anterior limb of internal capsule on FA skeleton (right)	25168	Diffusion MRI	2289
Mean MO in anterior limb of internal capsule on FA skeleton (left)	25169	Diffusion MRI	2289
Mean MO in posterior limb of internal capsule on FA skeleton (right)	25170	Diffusion MRI	2289
Mean MO in posterior limb of internal capsule on FA skeleton (left)	25171	Diffusion MRI	2289
Mean MO in retrolenticular part of internal capsule on FA skeleton (right)	25172	Diffusion MRI	2289
Mean MO in retrolenticular part of internal capsule on FA skeleton (left)	25173	Diffusion MRI	2289
Mean MO in anterior corona radiata on FA skeleton (right)	25174	Diffusion MRI	2289
Mean MO in anterior corona radiata on FA skeleton (left)	25175	Diffusion MRI	2289
Mean MO in superior corona radiata on FA skeleton (right)	25176	Diffusion MRI	2289
Mean MO in superior corona radiata on FA skeleton (left)	25177	Diffusion MRI	2289
Mean MO in posterior corona radiata on FA skeleton (right)	25178	Diffusion MRI	2289
Mean MO in posterior corona radiata on FA skeleton (left)	25179	Diffusion MRI	2289
Mean MO in posterior thalamic radiation on FA skeleton (right)	25180	Diffusion MRI	2289

Mean MO in posterior thalamic radiation on FA skeleton (left)	25181	Diffusion MRI	2289
Mean MO in sagittal stratum on FA skeleton (right)	25182	Diffusion MRI	2289
Mean MO in sagittal stratum on FA skeleton (left)	25183	Diffusion MRI	2289
Mean MO in external capsule on FA skeleton (right)	25184	Diffusion MRI	2289
Mean MO in external capsule on FA skeleton (left)	25185	Diffusion MRI	2289
Mean MO in cingulum cingulate gyrus on FA skeleton (right)	25186	Diffusion MRI	2289
Mean MO in cingulum cingulate gyrus on FA skeleton (left)	25187	Diffusion MRI	2289
Mean MO in cingulum hippocampus on FA skeleton (right)	25188	Diffusion MRI	2289
Mean MO in cingulum hippocampus on FA skeleton (left)	25189	Diffusion MRI	2289
Mean MO in fornix cres+stria terminalis on FA skeleton (right)	25190	Diffusion MRI	2289
Mean MO in fornix cres+stria terminalis on FA skeleton (left)	25191	Diffusion MRI	2289
Mean MO in superior longitudinal fasciculus on FA skeleton (right)	25192	Diffusion MRI	2289
Mean MO in superior longitudinal fasciculus on FA skeleton (left)	25193	Diffusion MRI	2289
Mean MO in superior fronto-occipital fasciculus on FA skeleton (right)	25194	Diffusion MRI	2289
Mean MO in superior fronto-occipital fasciculus on FA skeleton (left)	25195	Diffusion MRI	2289
Mean MO in uncinate fasciculus on FA skeleton (right)	25196	Diffusion MRI	2289
Mean MO in uncinate fasciculus on FA skeleton (left)	25197	Diffusion MRI	2289
Mean MO in tapetum on FA skeleton (right)	25198	Diffusion MRI	2289
Mean MO in tapetum on FA skeleton (left)	25199	Diffusion MRI	2289
Mean L1 in middle cerebellar peduncle on FA skeleton	25200	Diffusion MRI	2289
Mean L1 in pontine crossing tract on FA skeleton	25201	Diffusion MRI	2289
Mean L1 in genu of corpus callosum on FA skeleton	25202	Diffusion MRI	2289
Mean L1 in body of corpus callosum on FA skeleton	25203	Diffusion MRI	2289
Mean L1 in splenium of corpus callosum on FA skeleton	25204	Diffusion MRI	2289
Mean L1 in fornix on FA skeleton	25205	Diffusion MRI	2289
Mean L1 in corticospinal tract on FA skeleton (right)	25206	Diffusion MRI	2289
Mean L1 in corticospinal tract on FA skeleton (left)	25207	Diffusion MRI	2289
Mean L1 in medial lemniscus on FA skeleton (right)	25208	Diffusion MRI	2289
Mean L1 in medial lemniscus on FA skeleton (left)	25209	Diffusion MRI	2289
Mean L1 in inferior cerebellar peduncle on FA skeleton (right)	25210	Diffusion MRI	2289
Mean L1 in inferior cerebellar peduncle on FA skeleton (left)	25211	Diffusion MRI	2289
Mean L1 in superior cerebellar peduncle on FA skeleton (right)	25212	Diffusion MRI	2289
Mean L1 in superior cerebellar peduncle on FA skeleton (left)	25213	Diffusion MRI	2289
Mean L1 in cerebral peduncle on FA skeleton (right)	25214	Diffusion MRI	2289
Mean L1 in cerebral peduncle on FA skeleton (left)	25215	Diffusion MRI	2289
Mean L1 in anterior limb of internal capsule on FA skeleton (right)	25216	Diffusion MRI	2289
Mean L1 in anterior limb of internal capsule on FA skeleton (left)	25217	Diffusion MRI	2289
Mean L1 in posterior limb of internal capsule on FA skeleton (right)	25218	Diffusion MRI	2289
Mean L1 in posterior limb of internal capsule on FA skeleton (left)	25219	Diffusion MRI	2289

Mean L1 in retrolenticular part of internal capsule on FA skeleton (right)	25220	Diffusion MRI	2289
Mean L1 in retrolenticular part of internal capsule on FA skeleton (left)	25221	Diffusion MRI	2289
Mean L1 in anterior corona radiata on FA skeleton (right)	25222	Diffusion MRI	2289
Mean L1 in anterior corona radiata on FA skeleton (left)	25223	Diffusion MRI	2289
Mean L1 in superior corona radiata on FA skeleton (right)	25224	Diffusion MRI	2289
Mean L1 in superior corona radiata on FA skeleton (left)	25225	Diffusion MRI	2289
Mean L1 in posterior corona radiata on FA skeleton (right)	25226	Diffusion MRI	2289
Mean L1 in posterior corona radiata on FA skeleton (left)	25227	Diffusion MRI	2289
Mean L1 in posterior thalamic radiation on FA skeleton (right)	25228	Diffusion MRI	2289
Mean L1 in posterior thalamic radiation on FA skeleton (left)	25229	Diffusion MRI	2289
Mean L1 in sagittal stratum on FA skeleton (right)	25230	Diffusion MRI	2289
Mean L1 in sagittal stratum on FA skeleton (left)	25231	Diffusion MRI	2289
Mean L1 in external capsule on FA skeleton (right)	25232	Diffusion MRI	2289
Mean L1 in external capsule on FA skeleton (left)	25233	Diffusion MRI	2289
Mean L1 in cingulum cingulate gyrus on FA skeleton (right)	25234	Diffusion MRI	2289
Mean L1 in cingulum cingulate gyrus on FA skeleton (left)	25235	Diffusion MRI	2289
Mean L1 in cingulum hippocampus on FA skeleton (right)	25236	Diffusion MRI	2289
Mean L1 in cingulum hippocampus on FA skeleton (left)	25237	Diffusion MRI	2289
Mean L1 in fornix cres+stria terminalis on FA skeleton (right)	25238	Diffusion MRI	2289
Mean L1 in fornix cres+stria terminalis on FA skeleton (left)	25239	Diffusion MRI	2289
Mean L1 in superior longitudinal fasciculus on FA skeleton (right)	25240	Diffusion MRI	2289
Mean L1 in superior longitudinal fasciculus on FA skeleton (left)	25241	Diffusion MRI	2289
Mean L1 in superior fronto-occipital fasciculus on FA skeleton (right)	25242	Diffusion MRI	2289
Mean L1 in superior fronto-occipital fasciculus on FA skeleton (left)	25243	Diffusion MRI	2289
Mean L1 in uncinate fasciculus on FA skeleton (right)	25244	Diffusion MRI	2289
Mean L1 in uncinate fasciculus on FA skeleton (left)	25245	Diffusion MRI	2289
Mean L1 in tapetum on FA skeleton (right)	25246	Diffusion MRI	2289
Mean L1 in tapetum on FA skeleton (left)	25247	Diffusion MRI	2289
Mean L2 in middle cerebellar peduncle on FA skeleton	25248	Diffusion MRI	2289
Mean L2 in pontine crossing tract on FA skeleton	25249	Diffusion MRI	2289
Mean L2 in genu of corpus callosum on FA skeleton	25250	Diffusion MRI	2289
Mean L2 in body of corpus callosum on FA skeleton	25251	Diffusion MRI	2289
Mean L2 in splenium of corpus callosum on FA skeleton	25252	Diffusion MRI	2289
Mean L2 in fornix on FA skeleton	25253	Diffusion MRI	2289
Mean L2 in corticospinal tract on FA skeleton (right)	25254	Diffusion MRI	2289
Mean L2 in corticospinal tract on FA skeleton (left)	25255	Diffusion MRI	2289
Mean L2 in medial lemniscus on FA skeleton (right)	25256	Diffusion MRI	2289
Mean L2 in medial lemniscus on FA skeleton (left)	25257	Diffusion MRI	2289
Mean L2 in inferior cerebellar peduncle on FA skeleton (right)	25258	Diffusion MRI	2289
Mean L2 in inferior cerebellar peduncle on FA skeleton (left)	25259	Diffusion MRI	2289

Mean L2 in superior cerebellar peduncle on FA skeleton (right)	25260	Diffusion MRI	2289
Mean L2 in superior cerebellar peduncle on FA skeleton (left)	25261	Diffusion MRI	2289
Mean L2 in cerebral peduncle on FA skeleton (right)	25262	Diffusion MRI	2289
Mean L2 in cerebral peduncle on FA skeleton (left)	25263	Diffusion MRI	2289
Mean L2 in anterior limb of internal capsule on FA skeleton (right)	25264	Diffusion MRI	2289
Mean L2 in anterior limb of internal capsule on FA skeleton (left)	25265	Diffusion MRI	2289
Mean L2 in posterior limb of internal capsule on FA skeleton (right)	25266	Diffusion MRI	2289
Mean L2 in posterior limb of internal capsule on FA skeleton (left)	25267	Diffusion MRI	2289
Mean L2 in retrolenticular part of internal capsule on FA skeleton (right)	25268	Diffusion MRI	2289
Mean L2 in retrolenticular part of internal capsule on FA skeleton (left)	25269	Diffusion MRI	2289
Mean L2 in anterior corona radiata on FA skeleton (right)	25270	Diffusion MRI	2289
Mean L2 in anterior corona radiata on FA skeleton (left)	25271	Diffusion MRI	2289
Mean L2 in superior corona radiata on FA skeleton (right)	25272	Diffusion MRI	2289
Mean L2 in superior corona radiata on FA skeleton (left)	25273	Diffusion MRI	2289
Mean L2 in posterior corona radiata on FA skeleton (right)	25274	Diffusion MRI	2289
Mean L2 in posterior corona radiata on FA skeleton (left)	25275	Diffusion MRI	2289
Mean L2 in posterior thalamic radiation on FA skeleton (right)	25276	Diffusion MRI	2289
Mean L2 in posterior thalamic radiation on FA skeleton (left)	25277	Diffusion MRI	2289
Mean L2 in sagittal stratum on FA skeleton (right)	25278	Diffusion MRI	2289
Mean L2 in sagittal stratum on FA skeleton (left)	25279	Diffusion MRI	2289
Mean L2 in external capsule on FA skeleton (right)	25280	Diffusion MRI	2289
Mean L2 in external capsule on FA skeleton (left)	25281	Diffusion MRI	2289
Mean L2 in cingulum cingulate gyrus on FA skeleton (right)	25282	Diffusion MRI	2289
Mean L2 in cingulum cingulate gyrus on FA skeleton (left)	25283	Diffusion MRI	2289
Mean L2 in cingulum hippocampus on FA skeleton (right)	25284	Diffusion MRI	2289
Mean L2 in cingulum hippocampus on FA skeleton (left)	25285	Diffusion MRI	2289
Mean L2 in fornix cres+stria terminalis on FA skeleton (right)	25286	Diffusion MRI	2289
Mean L2 in fornix cres+stria terminalis on FA skeleton (left)	25287	Diffusion MRI	2289
Mean L2 in superior longitudinal fasciculus on FA skeleton (right)	25288	Diffusion MRI	2289
Mean L2 in superior longitudinal fasciculus on FA skeleton (left)	25289	Diffusion MRI	2289
Mean L2 in superior fronto-occipital fasciculus on FA skeleton (right)	25290	Diffusion MRI	2289
Mean L2 in superior fronto-occipital fasciculus on FA skeleton (left)	25291	Diffusion MRI	2289
Mean L2 in uncinate fasciculus on FA skeleton (right)	25292	Diffusion MRI	2289
Mean L2 in uncinate fasciculus on FA skeleton (left)	25293	Diffusion MRI	2289
Mean L2 in tapetum on FA skeleton (right)	25294	Diffusion MRI	2289
Mean L2 in tapetum on FA skeleton (left)	25295	Diffusion MRI	2289
Mean L3 in middle cerebellar peduncle on FA skeleton	25296	Diffusion MRI	2289
Mean L3 in pontine crossing tract on FA skeleton	25297	Diffusion MRI	2289

Mean L3 in genu of corpus callosum on FA skeleton	25298	Diffusion MRI	2289
Mean L3 in body of corpus callosum on FA skeleton	25299	Diffusion MRI	2289
Mean L3 in splenium of corpus callosum on FA skeleton	25300	Diffusion MRI	2289
Mean L3 in fornix on FA skeleton	25301	Diffusion MRI	2289
Mean L3 in corticospinal tract on FA skeleton (right)	25302	Diffusion MRI	2289
Mean L3 in corticospinal tract on FA skeleton (left)	25303	Diffusion MRI	2289
Mean L3 in medial lemniscus on FA skeleton (right)	25304	Diffusion MRI	2289
Mean L3 in medial lemniscus on FA skeleton (left)	25305	Diffusion MRI	2289
Mean L3 in inferior cerebellar peduncle on FA skeleton (right)	25306	Diffusion MRI	2289
Mean L3 in inferior cerebellar peduncle on FA skeleton (left)	25307	Diffusion MRI	2289
Mean L3 in superior cerebellar peduncle on FA skeleton (right)	25308	Diffusion MRI	2289
Mean L3 in superior cerebellar peduncle on FA skeleton (left)	25309	Diffusion MRI	2289
Mean L3 in cerebral peduncle on FA skeleton (right)	25310	Diffusion MRI	2289
Mean L3 in cerebral peduncle on FA skeleton (left)	25311	Diffusion MRI	2289
Mean L3 in anterior limb of internal capsule on FA skeleton (right)	25312	Diffusion MRI	2289
Mean L3 in anterior limb of internal capsule on FA skeleton (left)	25313	Diffusion MRI	2289
Mean L3 in posterior limb of internal capsule on FA skeleton (right)	25314	Diffusion MRI	2289
Mean L3 in posterior limb of internal capsule on FA skeleton (left)	25315	Diffusion MRI	2289
Mean L3 in retrolenticular part of internal capsule on FA skeleton (right)	25316	Diffusion MRI	2289
Mean L3 in retrolenticular part of internal capsule on FA skeleton (left)	25317	Diffusion MRI	2289
Mean L3 in anterior corona radiata on FA skeleton (right)	25318	Diffusion MRI	2289
Mean L3 in anterior corona radiata on FA skeleton (left)	25319	Diffusion MRI	2289
Mean L3 in superior corona radiata on FA skeleton (right)	25320	Diffusion MRI	2289
Mean L3 in superior corona radiata on FA skeleton (left)	25321	Diffusion MRI	2289
Mean L3 in posterior corona radiata on FA skeleton (right)	25322	Diffusion MRI	2289
Mean L3 in posterior corona radiata on FA skeleton (left)	25323	Diffusion MRI	2289
Mean L3 in posterior thalamic radiation on FA skeleton (right)	25324	Diffusion MRI	2289
Mean L3 in posterior thalamic radiation on FA skeleton (left)	25325	Diffusion MRI	2289
Mean L3 in sagittal stratum on FA skeleton (right)	25326	Diffusion MRI	2289
Mean L3 in sagittal stratum on FA skeleton (left)	25327	Diffusion MRI	2289
Mean L3 in external capsule on FA skeleton (right)	25328	Diffusion MRI	2289
Mean L3 in external capsule on FA skeleton (left)	25329	Diffusion MRI	2289
Mean L3 in cingulum cingulate gyrus on FA skeleton (right)	25330	Diffusion MRI	2289
Mean L3 in cingulum cingulate gyrus on FA skeleton (left)	25331	Diffusion MRI	2289
Mean L3 in cingulum hippocampus on FA skeleton (right)	25332	Diffusion MRI	2289
Mean L3 in cingulum hippocampus on FA skeleton (left)	25333	Diffusion MRI	2289
Mean L3 in fornix cres+stria terminalis on FA skeleton (right)	25334	Diffusion MRI	2289
Mean L3 in fornix cres+stria terminalis on FA skeleton (left)	25335	Diffusion MRI	2289
Mean L3 in superior longitudinal fasciculus on FA skeleton (right)	25336	Diffusion MRI	2289

Mean L3 in superior longitudinal fasciculus on FA skeleton (left)	25337	Diffusion MRI	2289
Mean L3 in superior fronto-occipital fasciculus on FA skeleton (right)	25338	Diffusion MRI	2289
Mean L3 in superior fronto-occipital fasciculus on FA skeleton (left)	25339	Diffusion MRI	2289
Mean L3 in uncinate fasciculus on FA skeleton (right)	25340	Diffusion MRI	2289
Mean L3 in uncinate fasciculus on FA skeleton (left)	25341	Diffusion MRI	2289
Mean L3 in tapetum on FA skeleton (right)	25342	Diffusion MRI	2289
Mean L3 in tapetum on FA skeleton (left)	25343	Diffusion MRI	2289
Mean ICVF in middle cerebellar peduncle on FA skeleton	25344	Diffusion MRI	2291
Mean ICVF in pontine crossing tract on FA skeleton	25345	Diffusion MRI	2291
Mean ICVF in genu of corpus callosum on FA skeleton	25346	Diffusion MRI	2291
Mean ICVF in body of corpus callosum on FA skeleton	25347	Diffusion MRI	2291
Mean ICVF in splenium of corpus callosum on FA skeleton	25348	Diffusion MRI	2291
Mean ICVF in fornix on FA skeleton	25349	Diffusion MRI	2291
Mean ICVF in corticospinal tract on FA skeleton (right)	25350	Diffusion MRI	2291
Mean ICVF in corticospinal tract on FA skeleton (left)	25351	Diffusion MRI	2291
Mean ICVF in medial lemniscus on FA skeleton (right)	25352	Diffusion MRI	2291
Mean ICVF in medial lemniscus on FA skeleton (left)	25353	Diffusion MRI	2291
Mean ICVF in inferior cerebellar peduncle on FA skeleton (right)	25354	Diffusion MRI	2291
Mean ICVF in inferior cerebellar peduncle on FA skeleton (left)	25355	Diffusion MRI	2291
Mean ICVF in superior cerebellar peduncle on FA skeleton (right)	25356	Diffusion MRI	2291
Mean ICVF in superior cerebellar peduncle on FA skeleton (left)	25357	Diffusion MRI	2291
Mean ICVF in cerebral peduncle on FA skeleton (right)	25358	Diffusion MRI	2291
Mean ICVF in cerebral peduncle on FA skeleton (left)	25359	Diffusion MRI	2291
Mean ICVF in anterior limb of internal capsule on FA skeleton (right)	25360	Diffusion MRI	2291
Mean ICVF in anterior limb of internal capsule on FA skeleton (left)	25361	Diffusion MRI	2291
Mean ICVF in posterior limb of internal capsule on FA skeleton (right)	25362	Diffusion MRI	2291
Mean ICVF in posterior limb of internal capsule on FA skeleton (left)	25363	Diffusion MRI	2291
Mean ICVF in retrolenticular part of internal capsule on FA skeleton (right)	25364	Diffusion MRI	2291
Mean ICVF in retrolenticular part of internal capsule on FA skeleton (left)	25365	Diffusion MRI	2291
Mean ICVF in anterior corona radiata on FA skeleton (right)	25366	Diffusion MRI	2291
Mean ICVF in anterior corona radiata on FA skeleton (left)	25367	Diffusion MRI	2291
Mean ICVF in superior corona radiata on FA skeleton (right)	25368	Diffusion MRI	2291
Mean ICVF in superior corona radiata on FA skeleton (left)	25369	Diffusion MRI	2291
Mean ICVF in posterior corona radiata on FA skeleton (right)	25370	Diffusion MRI	2291
Mean ICVF in posterior corona radiata on FA skeleton (left)	25371	Diffusion MRI	2291
Mean ICVF in posterior thalamic radiation on FA skeleton (right)	25372	Diffusion MRI	2291
Mean ICVF in posterior thalamic radiation on FA skeleton (left)	25373	Diffusion MRI	2291

Mean ICVF in sagittal stratum on FA skeleton (right)	25374	Diffusion MRI	2291
Mean ICVF in sagittal stratum on FA skeleton (left)	25375	Diffusion MRI	2291
Mean ICVF in external capsule on FA skeleton (right)	25376	Diffusion MRI	2291
Mean ICVF in external capsule on FA skeleton (left)	25377	Diffusion MRI	2291
Mean ICVF in cingulum cingulate gyrus on FA skeleton (right)	25378	Diffusion MRI	2291
Mean ICVF in cingulum cingulate gyrus on FA skeleton (left)	25379	Diffusion MRI	2291
Mean ICVF in cingulum hippocampus on FA skeleton (right)	25380	Diffusion MRI	2291
Mean ICVF in cingulum hippocampus on FA skeleton (left)	25381	Diffusion MRI	2291
Mean ICVF in fornix cres+stria terminalis on FA skeleton (right)	25382	Diffusion MRI	2291
Mean ICVF in fornix cres+stria terminalis on FA skeleton (left)	25383	Diffusion MRI	2291
Mean ICVF in superior longitudinal fasciculus on FA skeleton (right)	25384	Diffusion MRI	2291
Mean ICVF in superior longitudinal fasciculus on FA skeleton (left)	25385	Diffusion MRI	2291
Mean ICVF in superior fronto-occipital fasciculus on FA skeleton (right)	25386	Diffusion MRI	2291
Mean ICVF in superior fronto-occipital fasciculus on FA skeleton (left)	25387	Diffusion MRI	2291
Mean ICVF in uncinate fasciculus on FA skeleton (right)	25388	Diffusion MRI	2291
Mean ICVF in uncinate fasciculus on FA skeleton (left)	25389	Diffusion MRI	2291
Mean ICVF in tapetum on FA skeleton (right)	25390	Diffusion MRI	2291
Mean ICVF in tapetum on FA skeleton (left)	25391	Diffusion MRI	2291
Mean OD in middle cerebellar peduncle on FA skeleton	25392	Diffusion MRI	2291
Mean OD in pontine crossing tract on FA skeleton	25393	Diffusion MRI	2291
Mean OD in genu of corpus callosum on FA skeleton	25394	Diffusion MRI	2291
Mean OD in body of corpus callosum on FA skeleton	25395	Diffusion MRI	2291
Mean OD in splenium of corpus callosum on FA skeleton	25396	Diffusion MRI	2291
Mean OD in fornix on FA skeleton	25397	Diffusion MRI	2291
Mean OD in corticospinal tract on FA skeleton (right)	25398	Diffusion MRI	2291
Mean OD in corticospinal tract on FA skeleton (left)	25399	Diffusion MRI	2291
Mean OD in medial lemniscus on FA skeleton (right)	25400	Diffusion MRI	2291
Mean OD in medial lemniscus on FA skeleton (left)	25401	Diffusion MRI	2291
Mean OD in inferior cerebellar peduncle on FA skeleton (right)	25402	Diffusion MRI	2291
Mean OD in inferior cerebellar peduncle on FA skeleton (left)	25403	Diffusion MRI	2291
Mean OD in superior cerebellar peduncle on FA skeleton (right)	25404	Diffusion MRI	2291
Mean OD in superior cerebellar peduncle on FA skeleton (left)	25405	Diffusion MRI	2291
Mean OD in cerebral peduncle on FA skeleton (right)	25406	Diffusion MRI	2291
Mean OD in cerebral peduncle on FA skeleton (left)	25407	Diffusion MRI	2291
Mean OD in anterior limb of internal capsule on FA skeleton (right)	25408	Diffusion MRI	2291
Mean OD in anterior limb of internal capsule on FA skeleton (left)	25409	Diffusion MRI	2291
Mean OD in posterior limb of internal capsule on FA skeleton (right)	25410	Diffusion MRI	2291

Mean OD in posterior limb of internal capsule on FA skeleton (left)	25411	Diffusion MRI	2291
Mean OD in retrolenticular part of internal capsule on FA skeleton (right)	25412	Diffusion MRI	2291
Mean OD in retrolenticular part of internal capsule on FA skeleton (left)	25413	Diffusion MRI	2291
Mean OD in anterior corona radiata on FA skeleton (right)	25414	Diffusion MRI	2291
Mean OD in anterior corona radiata on FA skeleton (left)	25415	Diffusion MRI	2291
Mean OD in superior corona radiata on FA skeleton (right)	25416	Diffusion MRI	2291
Mean OD in superior corona radiata on FA skeleton (left)	25417	Diffusion MRI	2291
Mean OD in posterior corona radiata on FA skeleton (right)	25418	Diffusion MRI	2291
Mean OD in posterior corona radiata on FA skeleton (left)	25419	Diffusion MRI	2291
Mean OD in posterior thalamic radiation on FA skeleton (right)	25420	Diffusion MRI	2291
Mean OD in posterior thalamic radiation on FA skeleton (left)	25421	Diffusion MRI	2291
Mean OD in sagittal stratum on FA skeleton (right)	25422	Diffusion MRI	2291
Mean OD in sagittal stratum on FA skeleton (left)	25423	Diffusion MRI	2291
Mean OD in external capsule on FA skeleton (right)	25424	Diffusion MRI	2291
Mean OD in external capsule on FA skeleton (left)	25425	Diffusion MRI	2291
Mean OD in cingulum cingulate gyrus on FA skeleton (right)	25426	Diffusion MRI	2291
Mean OD in cingulum cingulate gyrus on FA skeleton (left)	25427	Diffusion MRI	2291
Mean OD in cingulum hippocampus on FA skeleton (right)	25428	Diffusion MRI	2291
Mean OD in cingulum hippocampus on FA skeleton (left)	25429	Diffusion MRI	2291
Mean OD in fornix cres+stria terminalis on FA skeleton (right)	25430	Diffusion MRI	2291
Mean OD in fornix cres+stria terminalis on FA skeleton (left)	25431	Diffusion MRI	2291
Mean OD in superior longitudinal fasciculus on FA skeleton (right)	25432	Diffusion MRI	2291
Mean OD in superior longitudinal fasciculus on FA skeleton (left)	25433	Diffusion MRI	2291
Mean OD in superior fronto-occipital fasciculus on FA skeleton (right)	25434	Diffusion MRI	2291
Mean OD in superior fronto-occipital fasciculus on FA skeleton (left)	25435	Diffusion MRI	2291
Mean OD in uncinate fasciculus on FA skeleton (right)	25436	Diffusion MRI	2291
Mean OD in uncinate fasciculus on FA skeleton (left)	25437	Diffusion MRI	2291
Mean OD in tapetum on FA skeleton (right)	25438	Diffusion MRI	2291
Mean OD in tapetum on FA skeleton (left)	25439	Diffusion MRI	2291
Mean ISOVF in middle cerebellar peduncle on FA skeleton	25440	Diffusion MRI	2291
Mean ISOVF in pontine crossing tract on FA skeleton	25441	Diffusion MRI	2291
Mean ISOVF in genu of corpus callosum on FA skeleton	25442	Diffusion MRI	2291
Mean ISOVF in body of corpus callosum on FA skeleton	25443	Diffusion MRI	2291
Mean ISOVF in splenium of corpus callosum on FA skeleton	25444	Diffusion MRI	2291
Mean ISOVF in fornix on FA skeleton	25445	Diffusion MRI	2291
Mean ISOVF in corticospinal tract on FA skeleton (right)	25446	Diffusion MRI	2291
Mean ISOVF in corticospinal tract on FA skeleton (left)	25447	Diffusion MRI	2291
Mean ISOVF in medial lemniscus on FA skeleton (right)	25448	Diffusion MRI	2291
Mean ISOVF in medial lemniscus on FA skeleton (left)	25449	Diffusion MRI	2291

Mean ISOVF in inferior cerebellar peduncle on FA skeleton (right)	25450	Diffusion MRI	2291
Mean ISOVF in inferior cerebellar peduncle on FA skeleton (left)	25451	Diffusion MRI	2291
Mean ISOVF in superior cerebellar peduncle on FA skeleton (right)	25452	Diffusion MRI	2291
Mean ISOVF in superior cerebellar peduncle on FA skeleton (left)	25453	Diffusion MRI	2291
Mean ISOVF in cerebral peduncle on FA skeleton (right)	25454	Diffusion MRI	2291
Mean ISOVF in cerebral peduncle on FA skeleton (left)	25455	Diffusion MRI	2291
Mean ISOVF in anterior limb of internal capsule on FA skeleton (right)	25456	Diffusion MRI	2291
Mean ISOVF in anterior limb of internal capsule on FA skeleton (left)	25457	Diffusion MRI	2291
Mean ISOVF in posterior limb of internal capsule on FA skeleton (right)	25458	Diffusion MRI	2291
Mean ISOVF in posterior limb of internal capsule on FA skeleton (left)	25459	Diffusion MRI	2291
Mean ISOVF in retrolenticular part of internal capsule on FA skeleton (right)	25460	Diffusion MRI	2291
Mean ISOVF in retrolenticular part of internal capsule on FA skeleton (left)	25461	Diffusion MRI	2291
Mean ISOVF in anterior corona radiata on FA skeleton (right)	25462	Diffusion MRI	2291
Mean ISOVF in anterior corona radiata on FA skeleton (left)	25463	Diffusion MRI	2291
Mean ISOVF in superior corona radiata on FA skeleton (right)	25464	Diffusion MRI	2291
Mean ISOVF in superior corona radiata on FA skeleton (left)	25465	Diffusion MRI	2291
Mean ISOVF in posterior corona radiata on FA skeleton (right)	25466	Diffusion MRI	2291
Mean ISOVF in posterior corona radiata on FA skeleton (left)	25467	Diffusion MRI	2291
Mean ISOVF in posterior thalamic radiation on FA skeleton (right)	25468	Diffusion MRI	2291
Mean ISOVF in posterior thalamic radiation on FA skeleton (left)	25469	Diffusion MRI	2291
Mean ISOVF in sagittal stratum on FA skeleton (right)	25470	Diffusion MRI	2291
Mean ISOVF in sagittal stratum on FA skeleton (left)	25471	Diffusion MRI	2291
Mean ISOVF in external capsule on FA skeleton (right)	25472	Diffusion MRI	2291
Mean ISOVF in external capsule on FA skeleton (left)	25473	Diffusion MRI	2291
Mean ISOVF in cingulum cingulate gyrus on FA skeleton (right)	25474	Diffusion MRI	2291
Mean ISOVF in cingulum cingulate gyrus on FA skeleton (left)	25475	Diffusion MRI	2291
Mean ISOVF in cingulum hippocampus on FA skeleton (right)	25476	Diffusion MRI	2291
Mean ISOVF in cingulum hippocampus on FA skeleton (left)	25477	Diffusion MRI	2291
Mean ISOVF in fornix cres+stria terminalis on FA skeleton (right)	25478	Diffusion MRI	2291
Mean ISOVF in fornix cres+stria terminalis on FA skeleton (left)	25479	Diffusion MRI	2291
Mean ISOVF in superior longitudinal fasciculus on FA skeleton (right)	25480	Diffusion MRI	2291
Mean ISOVF in superior longitudinal fasciculus on FA skeleton (left)	25481	Diffusion MRI	2291
Mean ISOVF in superior fronto-occipital fasciculus on FA skeleton (right)	25482	Diffusion MRI	2291
Mean ISOVF in superior fronto-occipital fasciculus on FA skeleton (left)	25483	Diffusion MRI	2291

Mean ISOVF in uncinate fasciculus on FA skeleton (right)	25484	Diffusion MRI	2291
Mean ISOVF in uncinate fasciculus on FA skeleton (left)	25485	Diffusion MRI	2291
Mean ISOVF in tapetum on FA skeleton (right)	25486	Diffusion MRI	2291
Mean ISOVF in tapetum on FA skeleton (left)	25487	Diffusion MRI	2291
Weighted-mean FA in tract acoustic radiation (left)	25488	Diffusion MRI	2291
Weighted-mean FA in tract acoustic radiation (right)	25489	Diffusion MRI	2291
Weighted-mean FA in tract anterior thalamic radiation (left)	25490	Diffusion MRI	2291
Weighted-mean FA in tract anterior thalamic radiation (right)	25491	Diffusion MRI	2291
Weighted-mean FA in tract cingulate gyrus part of cingulum (left)	25492	Diffusion MRI	2291
Weighted-mean FA in tract cingulate gyrus part of cingulum (right)	25493	Diffusion MRI	2291
Weighted-mean FA in tract parahippocampal part of cingulum (left)	25494	Diffusion MRI	2291
Weighted-mean FA in tract parahippocampal part of cingulum (right)	25495	Diffusion MRI	2291
Weighted-mean FA in tract corticospinal tract (left)	25496	Diffusion MRI	2291
Weighted-mean FA in tract corticospinal tract (right)	25497	Diffusion MRI	2291
Weighted-mean FA in tract forceps major	25498	Diffusion MRI	2291
Weighted-mean FA in tract forceps minor	25499	Diffusion MRI	2291
Weighted-mean FA in tract inferior fronto-occipital fasciculus (left)	25500	Diffusion MRI	2291
Weighted-mean FA in tract inferior fronto-occipital fasciculus (right)	25501	Diffusion MRI	2291
Weighted-mean FA in tract inferior longitudinal fasciculus (left)	25502	Diffusion MRI	2291
Weighted-mean FA in tract inferior longitudinal fasciculus (right)	25503	Diffusion MRI	2291
Weighted-mean FA in tract middle cerebellar peduncle	25504	Diffusion MRI	2291
Weighted-mean FA in tract medial lemniscus (left)	25505	Diffusion MRI	2291
Weighted-mean FA in tract medial lemniscus (right)	25506	Diffusion MRI	2291
Weighted-mean FA in tract posterior thalamic radiation (left)	25507	Diffusion MRI	2291
Weighted-mean FA in tract posterior thalamic radiation (right)	25508	Diffusion MRI	2291
Weighted-mean FA in tract superior longitudinal fasciculus (left)	25509	Diffusion MRI	2291
Weighted-mean FA in tract superior longitudinal fasciculus (right)	25510	Diffusion MRI	2291
Weighted-mean FA in tract superior thalamic radiation (left)	25511	Diffusion MRI	2291
Weighted-mean FA in tract superior thalamic radiation (right)	25512	Diffusion MRI	2291
Weighted-mean FA in tract uncinate fasciculus (left)	25513	Diffusion MRI	2291
Weighted-mean FA in tract uncinate fasciculus (right)	25514	Diffusion MRI	2291
Weighted-mean MD in tract acoustic radiation (left)	25515	Diffusion MRI	2291
Weighted-mean MD in tract acoustic radiation (right)	25516	Diffusion MRI	2291
Weighted-mean MD in tract anterior thalamic radiation (left)	25517	Diffusion MRI	2291
Weighted-mean MD in tract anterior thalamic radiation (right)	25518	Diffusion MRI	2291
Weighted-mean MD in tract cingulate gyrus part of cingulum (left)	25519	Diffusion MRI	2291
Weighted-mean MD in tract cingulate gyrus part of cingulum (right)	25520	Diffusion MRI	2291
Weighted-mean MD in tract parahippocampal part of cingulum (left)	25521	Diffusion MRI	2291

Weighted-mean MD in tract parahippocampal part of cingulum (right)	25522	Diffusion MRI	2291
Weighted-mean MD in tract corticospinal tract (left)	25523	Diffusion MRI	2291
Weighted-mean MD in tract corticospinal tract (right)	25524	Diffusion MRI	2291
Weighted-mean MD in tract forceps major	25525	Diffusion MRI	2291
Weighted-mean MD in tract forceps minor	25526	Diffusion MRI	2291
Weighted-mean MD in tract inferior fronto-occipital fasciculus (left)	25527	Diffusion MRI	2291
Weighted-mean MD in tract inferior fronto-occipital fasciculus (right)	25528	Diffusion MRI	2291
Weighted-mean MD in tract inferior longitudinal fasciculus (left)	25529	Diffusion MRI	2291
Weighted-mean MD in tract inferior longitudinal fasciculus (right)	25530	Diffusion MRI	2291
Weighted-mean MD in tract middle cerebellar peduncle	25531	Diffusion MRI	2291
Weighted-mean MD in tract medial lemniscus (left)	25532	Diffusion MRI	2291
Weighted-mean MD in tract medial lemniscus (right)	25533	Diffusion MRI	2291
Weighted-mean MD in tract posterior thalamic radiation (left)	25534	Diffusion MRI	2291
Weighted-mean MD in tract posterior thalamic radiation (right)	25535	Diffusion MRI	2291
Weighted-mean MD in tract superior longitudinal fasciculus (left)	25536	Diffusion MRI	2291
Weighted-mean MD in tract superior longitudinal fasciculus (right)	25537	Diffusion MRI	2291
Weighted-mean MD in tract superior thalamic radiation (left)	25538	Diffusion MRI	2291
Weighted-mean MD in tract superior thalamic radiation (right)	25539	Diffusion MRI	2291
Weighted-mean MD in tract uncinate fasciculus (left)	25540	Diffusion MRI	2291
Weighted-mean MD in tract uncinate fasciculus (right)	25541	Diffusion MRI	2291
Weighted-mean MO in tract acoustic radiation (left)	25542	Diffusion MRI	2291
Weighted-mean MO in tract acoustic radiation (right)	25543	Diffusion MRI	2291
Weighted-mean MO in tract anterior thalamic radiation (left)	25544	Diffusion MRI	2291
Weighted-mean MO in tract anterior thalamic radiation (right)	25545	Diffusion MRI	2291
Weighted-mean MO in tract cingulate gyrus part of cingulum (left)	25546	Diffusion MRI	2291
Weighted-mean MO in tract cingulate gyrus part of cingulum (right)	25547	Diffusion MRI	2291
Weighted-mean MO in tract parahippocampal part of cingulum (left)	25548	Diffusion MRI	2291
Weighted-mean MO in tract parahippocampal part of cingulum (right)	25549	Diffusion MRI	2291
Weighted-mean MO in tract corticospinal tract (left)	25550	Diffusion MRI	2291
Weighted-mean MO in tract corticospinal tract (right)	25551	Diffusion MRI	2291
Weighted-mean MO in tract forceps major	25552	Diffusion MRI	2291
Weighted-mean MO in tract forceps minor	25553	Diffusion MRI	2291
Weighted-mean MO in tract inferior fronto-occipital fasciculus (left)	25554	Diffusion MRI	2291
Weighted-mean MO in tract inferior fronto-occipital fasciculus (right)	25555	Diffusion MRI	2291
Weighted-mean MO in tract inferior longitudinal fasciculus (left)	25556	Diffusion MRI	2291
Weighted-mean MO in tract inferior longitudinal fasciculus (right)	25557	Diffusion MRI	2291

Weighted-mean MO in tract middle cerebellar peduncle	25558	Diffusion MRI	2291
Weighted-mean MO in tract medial lemniscus (left)	25559	Diffusion MRI	2291
Weighted-mean MO in tract medial lemniscus (right)	25560	Diffusion MRI	2291
Weighted-mean MO in tract posterior thalamic radiation (left)	25561	Diffusion MRI	2291
Weighted-mean MO in tract posterior thalamic radiation (right)	25562	Diffusion MRI	2291
Weighted-mean MO in tract superior longitudinal fasciculus (left)	25563	Diffusion MRI	2291
Weighted-mean MO in tract superior longitudinal fasciculus (right)	25564	Diffusion MRI	2291
Weighted-mean MO in tract superior thalamic radiation (left)	25565	Diffusion MRI	2291
Weighted-mean MO in tract superior thalamic radiation (right)	25566	Diffusion MRI	2291
Weighted-mean MO in tract uncinate fasciculus (left)	25567	Diffusion MRI	2291
Weighted-mean MO in tract uncinate fasciculus (right)	25568	Diffusion MRI	2291
Weighted-mean L1 in tract acoustic radiation (left)	25569	Diffusion MRI	2291
Weighted-mean L1 in tract acoustic radiation (right)	25570	Diffusion MRI	2291
Weighted-mean L1 in tract anterior thalamic radiation (left)	25571	Diffusion MRI	2291
Weighted-mean L1 in tract anterior thalamic radiation (right)	25572	Diffusion MRI	2291
Weighted-mean L1 in tract cingulate gyrus part of cingulum (left)	25573	Diffusion MRI	2291
Weighted-mean L1 in tract cingulate gyrus part of cingulum (right)	25574	Diffusion MRI	2291
Weighted-mean L1 in tract parahippocampal part of cingulum (left)	25575	Diffusion MRI	2291
Weighted-mean L1 in tract parahippocampal part of cingulum (right)	25576	Diffusion MRI	2291
Weighted-mean L1 in tract corticospinal tract (left)	25577	Diffusion MRI	2291
Weighted-mean L1 in tract corticospinal tract (right)	25578	Diffusion MRI	2291
Weighted-mean L1 in tract forceps major	25579	Diffusion MRI	2291
Weighted-mean L1 in tract forceps minor	25580	Diffusion MRI	2291
Weighted-mean L1 in tract inferior fronto-occipital fasciculus (left)	25581	Diffusion MRI	2291
Weighted-mean L1 in tract inferior fronto-occipital fasciculus (right)	25582	Diffusion MRI	2291
Weighted-mean L1 in tract inferior longitudinal fasciculus (left)	25583	Diffusion MRI	2291
Weighted-mean L1 in tract inferior longitudinal fasciculus (right)	25584	Diffusion MRI	2291
Weighted-mean L1 in tract middle cerebellar peduncle	25585	Diffusion MRI	2291
Weighted-mean L1 in tract medial lemniscus (left)	25586	Diffusion MRI	2291
Weighted-mean L1 in tract medial lemniscus (right)	25587	Diffusion MRI	2291
Weighted-mean L1 in tract posterior thalamic radiation (left)	25588	Diffusion MRI	2291
Weighted-mean L1 in tract posterior thalamic radiation (right)	25589	Diffusion MRI	2291
Weighted-mean L1 in tract superior longitudinal fasciculus (left)	25590	Diffusion MRI	2291
Weighted-mean L1 in tract superior longitudinal fasciculus (right)	25591	Diffusion MRI	2291
Weighted-mean L1 in tract superior thalamic radiation (left)	25592	Diffusion MRI	2291
Weighted-mean L1 in tract superior thalamic radiation (right)	25593	Diffusion MRI	2291
Weighted-mean L1 in tract uncinate fasciculus (left)	25594	Diffusion MRI	2291

Weighted-mean L1 in tract uncinate fasciculus (right)	25595	Diffusion MRI	2291
Weighted-mean L2 in tract acoustic radiation (left)	25596	Diffusion MRI	2291
Weighted-mean L2 in tract acoustic radiation (right)	25597	Diffusion MRI	2291
Weighted-mean L2 in tract anterior thalamic radiation (left)	25598	Diffusion MRI	2291
Weighted-mean L2 in tract anterior thalamic radiation (right)	25599	Diffusion MRI	2291
Weighted-mean L2 in tract cingulate gyrus part of cingulum (left)	25600	Diffusion MRI	2291
Weighted-mean L2 in tract cingulate gyrus part of cingulum (right)	25601	Diffusion MRI	2291
Weighted-mean L2 in tract parahippocampal part of cingulum (left)	25602	Diffusion MRI	2291
Weighted-mean L2 in tract parahippocampal part of cingulum (right)	25603	Diffusion MRI	2291
Weighted-mean L2 in tract corticospinal tract (left)	25604	Diffusion MRI	2291
Weighted-mean L2 in tract corticospinal tract (right)	25605	Diffusion MRI	2291
Weighted-mean L2 in tract forceps major	25606	Diffusion MRI	2291
Weighted-mean L2 in tract forceps minor	25607	Diffusion MRI	2291
Weighted-mean L2 in tract inferior fronto-occipital fasciculus (left)	25608	Diffusion MRI	2291
Weighted-mean L2 in tract inferior fronto-occipital fasciculus (right)	25609	Diffusion MRI	2291
Weighted-mean L2 in tract inferior longitudinal fasciculus (left)	25610	Diffusion MRI	2291
Weighted-mean L2 in tract inferior longitudinal fasciculus (right)	25611	Diffusion MRI	2291
Weighted-mean L2 in tract middle cerebellar peduncle	25612	Diffusion MRI	2291
Weighted-mean L2 in tract medial lemniscus (left)	25613	Diffusion MRI	2291
Weighted-mean L2 in tract medial lemniscus (right)	25614	Diffusion MRI	2291
Weighted-mean L2 in tract posterior thalamic radiation (left)	25615	Diffusion MRI	2291
Weighted-mean L2 in tract posterior thalamic radiation (right)	25616	Diffusion MRI	2291
Weighted-mean L2 in tract superior longitudinal fasciculus (left)	25617	Diffusion MRI	2291
Weighted-mean L2 in tract superior longitudinal fasciculus (right)	25618	Diffusion MRI	2291
Weighted-mean L2 in tract superior thalamic radiation (left)	25619	Diffusion MRI	2291
Weighted-mean L2 in tract superior thalamic radiation (right)	25620	Diffusion MRI	2291
Weighted-mean L2 in tract uncinate fasciculus (left)	25621	Diffusion MRI	2291
Weighted-mean L2 in tract uncinate fasciculus (right)	25622	Diffusion MRI	2291
Weighted-mean L3 in tract acoustic radiation (left)	25623	Diffusion MRI	2291
Weighted-mean L3 in tract acoustic radiation (right)	25624	Diffusion MRI	2291
Weighted-mean L3 in tract anterior thalamic radiation (left)	25625	Diffusion MRI	2291
Weighted-mean L3 in tract anterior thalamic radiation (right)	25626	Diffusion MRI	2291
Weighted-mean L3 in tract cingulate gyrus part of cingulum (left)	25627	Diffusion MRI	2291
Weighted-mean L3 in tract cingulate gyrus part of cingulum (right)	25628	Diffusion MRI	2291
Weighted-mean L3 in tract parahippocampal part of cingulum (left)	25629	Diffusion MRI	2291
Weighted-mean L3 in tract parahippocampal part of cingulum (right)	25630	Diffusion MRI	2291
Weighted-mean L3 in tract corticospinal tract (left)	25631	Diffusion MRI	2291

Weighted-mean L3 in tract corticospinal tract (right)	25632	Diffusion MRI	2291
Weighted-mean L3 in tract forceps major	25633	Diffusion MRI	2291
Weighted-mean L3 in tract forceps minor	25634	Diffusion MRI	2291
Weighted-mean L3 in tract inferior fronto-occipital fasciculus (left)	25635	Diffusion MRI	2291
Weighted-mean L3 in tract inferior fronto-occipital fasciculus (right)	25636	Diffusion MRI	2291
Weighted-mean L3 in tract inferior longitudinal fasciculus (left)	25637	Diffusion MRI	2291
Weighted-mean L3 in tract inferior longitudinal fasciculus (right)	25638	Diffusion MRI	2291
Weighted-mean L3 in tract middle cerebellar peduncle	25639	Diffusion MRI	2291
Weighted-mean L3 in tract medial lemniscus (left)	25640	Diffusion MRI	2291
Weighted-mean L3 in tract medial lemniscus (right)	25641	Diffusion MRI	2291
Weighted-mean L3 in tract posterior thalamic radiation (left)	25642	Diffusion MRI	2291
Weighted-mean L3 in tract posterior thalamic radiation (right)	25643	Diffusion MRI	2291
Weighted-mean L3 in tract superior longitudinal fasciculus (left)	25644	Diffusion MRI	2291
Weighted-mean L3 in tract superior longitudinal fasciculus (right)	25645	Diffusion MRI	2291
Weighted-mean L3 in tract superior thalamic radiation (left)	25646	Diffusion MRI	2291
Weighted-mean L3 in tract superior thalamic radiation (right)	25647	Diffusion MRI	2291
Weighted-mean L3 in tract uncinate fasciculus (left)	25648	Diffusion MRI	2291
Weighted-mean L3 in tract uncinate fasciculus (right)	25649	Diffusion MRI	2291
Weighted-mean ICVF in tract acoustic radiation (left)	25650	Diffusion MRI	2292
Weighted-mean ICVF in tract acoustic radiation (right)	25651	Diffusion MRI	2292
Weighted-mean ICVF in tract anterior thalamic radiation (left)	25652	Diffusion MRI	2292
Weighted-mean ICVF in tract anterior thalamic radiation (right)	25653	Diffusion MRI	2292
Weighted-mean ICVF in tract cingulate gyrus part of cingulum (left)	25654	Diffusion MRI	2292
Weighted-mean ICVF in tract cingulate gyrus part of cingulum (right)	25655	Diffusion MRI	2292
Weighted-mean ICVF in tract parahippocampal part of cingulum (left)	25656	Diffusion MRI	2292
Weighted-mean ICVF in tract parahippocampal part of cingulum (right)	25657	Diffusion MRI	2292
Weighted-mean ICVF in tract corticospinal tract (left)	25658	Diffusion MRI	2292
Weighted-mean ICVF in tract corticospinal tract (right)	25659	Diffusion MRI	2292
Weighted-mean ICVF in tract forceps major	25660	Diffusion MRI	2292
Weighted-mean ICVF in tract forceps minor	25661	Diffusion MRI	2292
Weighted-mean ICVF in tract inferior fronto-occipital fasciculus (left)	25662	Diffusion MRI	2292
Weighted-mean ICVF in tract inferior fronto-occipital fasciculus (right)	25663	Diffusion MRI	2292
Weighted-mean ICVF in tract inferior longitudinal fasciculus (left)	25664	Diffusion MRI	2292
Weighted-mean ICVF in tract inferior longitudinal fasciculus (right)	25665	Diffusion MRI	2292
Weighted-mean ICVF in tract middle cerebellar peduncle	25666	Diffusion MRI	2292
Weighted-mean ICVF in tract medial lemniscus (left)	25667	Diffusion MRI	2292
Weighted-mean ICVF in tract medial lemniscus (right)	25668	Diffusion MRI	2292

Weighted-mean ICVF in tract posterior thalamic radiation (left)	25669	Diffusion MRI	2292
Weighted-mean ICVF in tract posterior thalamic radiation (right)	25670	Diffusion MRI	2292
Weighted-mean ICVF in tract superior longitudinal fasciculus (left)	25671	Diffusion MRI	2292
Weighted-mean ICVF in tract superior longitudinal fasciculus (right)	25672	Diffusion MRI	2292
Weighted-mean ICVF in tract superior thalamic radiation (left)	25673	Diffusion MRI	2292
Weighted-mean ICVF in tract superior thalamic radiation (right)	25674	Diffusion MRI	2292
Weighted-mean ICVF in tract uncinate fasciculus (left)	25675	Diffusion MRI	2292
Weighted-mean ICVF in tract uncinate fasciculus (right)	25676	Diffusion MRI	2292
Weighted-mean OD in tract acoustic radiation (left)	25677	Diffusion MRI	2292
Weighted-mean OD in tract acoustic radiation (right)	25678	Diffusion MRI	2292
Weighted-mean OD in tract anterior thalamic radiation (left)	25679	Diffusion MRI	2292
Weighted-mean OD in tract anterior thalamic radiation (right)	25680	Diffusion MRI	2292
Weighted-mean OD in tract cingulate gyrus part of cingulum (left)	25681	Diffusion MRI	2292
Weighted-mean OD in tract cingulate gyrus part of cingulum (right)	25682	Diffusion MRI	2292
Weighted-mean OD in tract parahippocampal part of cingulum (left)	25683	Diffusion MRI	2292
Weighted-mean OD in tract parahippocampal part of cingulum (right)	25684	Diffusion MRI	2292
Weighted-mean OD in tract corticospinal tract (left)	25685	Diffusion MRI	2292
Weighted-mean OD in tract corticospinal tract (right)	25686	Diffusion MRI	2292
Weighted-mean OD in tract forceps major	25687	Diffusion MRI	2292
Weighted-mean OD in tract forceps minor	25688	Diffusion MRI	2292
Weighted-mean OD in tract inferior fronto-occipital fasciculus (left)	25689	Diffusion MRI	2292
Weighted-mean OD in tract inferior fronto-occipital fasciculus (right)	25690	Diffusion MRI	2292
Weighted-mean OD in tract inferior longitudinal fasciculus (left)	25691	Diffusion MRI	2292
Weighted-mean OD in tract inferior longitudinal fasciculus (right)	25692	Diffusion MRI	2292
Weighted-mean OD in tract middle cerebellar peduncle	25693	Diffusion MRI	2292
Weighted-mean OD in tract medial lemniscus (left)	25694	Diffusion MRI	2292
Weighted-mean OD in tract medial lemniscus (right)	25695	Diffusion MRI	2292
Weighted-mean OD in tract posterior thalamic radiation (left)	25696	Diffusion MRI	2292
Weighted-mean OD in tract posterior thalamic radiation (right)	25697	Diffusion MRI	2292
Weighted-mean OD in tract superior longitudinal fasciculus (left)	25698	Diffusion MRI	2292
Weighted-mean OD in tract superior longitudinal fasciculus (right)	25699	Diffusion MRI	2292
Weighted-mean OD in tract superior thalamic radiation (left)	25700	Diffusion MRI	2292
Weighted-mean OD in tract superior thalamic radiation (right)	25701	Diffusion MRI	2292
Weighted-mean OD in tract uncinate fasciculus (left)	25702	Diffusion MRI	2292
Weighted-mean OD in tract uncinate fasciculus (right)	25703	Diffusion MRI	2292
Weighted-mean ISOVF in tract acoustic radiation (left)	25704	Diffusion MRI	2292

Weighted-mean ISOVF in tract acoustic radiation (right)	25705	Diffusion MRI	2292
Weighted-mean ISOVF in tract anterior thalamic radiation (left)	25706	Diffusion MRI	2292
Weighted-mean ISOVF in tract anterior thalamic radiation (right)	25707	Diffusion MRI	2292
Weighted-mean ISOVF in tract cingulate gyrus part of cingulum (left)	25708	Diffusion MRI	2292
Weighted-mean ISOVF in tract cingulate gyrus part of cingulum (right)	25709	Diffusion MRI	2292
Weighted-mean ISOVF in tract parahippocampal part of cingulum (left)	25710	Diffusion MRI	2292
Weighted-mean ISOVF in tract parahippocampal part of cingulum (right)	25711	Diffusion MRI	2292
Weighted-mean ISOVF in tract corticospinal tract (left)	25712	Diffusion MRI	2292
Weighted-mean ISOVF in tract corticospinal tract (right)	25713	Diffusion MRI	2292
Weighted-mean ISOVF in tract forceps major	25714	Diffusion MRI	2292
Weighted-mean ISOVF in tract forceps minor	25715	Diffusion MRI	2292
Weighted-mean ISOVF in tract inferior fronto-occipital fasciculus (left)	25716	Diffusion MRI	2292
Weighted-mean ISOVF in tract inferior fronto-occipital fasciculus (right)	25717	Diffusion MRI	2292
Weighted-mean ISOVF in tract inferior longitudinal fasciculus (left)	25718	Diffusion MRI	2292
Weighted-mean ISOVF in tract inferior longitudinal fasciculus (right)	25719	Diffusion MRI	2292
Weighted-mean ISOVF in tract middle cerebellar peduncle	25720	Diffusion MRI	2292
Weighted-mean ISOVF in tract medial lemniscus (left)	25721	Diffusion MRI	2292
Weighted-mean ISOVF in tract medial lemniscus (right)	25722	Diffusion MRI	2292
Weighted-mean ISOVF in tract posterior thalamic radiation (left)	25723	Diffusion MRI	2292
Weighted-mean ISOVF in tract posterior thalamic radiation (right)	25724	Diffusion MRI	2292
Weighted-mean ISOVF in tract superior longitudinal fasciculus (left)	25725	Diffusion MRI	2292
Weighted-mean ISOVF in tract superior longitudinal fasciculus (right)	25726	Diffusion MRI	2292
Weighted-mean ISOVF in tract superior thalamic radiation (left)	25727	Diffusion MRI	2292
Weighted-mean ISOVF in tract superior thalamic radiation (right)	25728	Diffusion MRI	2292
Weighted-mean ISOVF in tract uncinate fasciculus (left)	25729	Diffusion MRI	2292
Weighted-mean ISOVF in tract uncinate fasciculus (right)	25730	Diffusion MRI	2292
rfMRI partial correlation matrix, dimension 25	25752*	Resting-state fMRI	1888
Median BOLD effect (in group-defined mask) for shapes activation	25040	Task fMRI	7373
Median z-statistic (in group-defined mask) for shapes activation	25042	Task fMRI	7373
Median BOLD effect (in group-defined mask) for faces activation	25044	Task fMRI	7373
Median z-statistic (in group-defined mask) for faces activation	25046	Task fMRI	7373
Median BOLD effect (in group-defined mask) for faces-shapes contrast	25048	Task fMRI	7373
Median BOLD effect (in group-defined amygdala activation mask) for faces-shapes contrast	25052	Task fMRI	7373

Median z-statistic (in group-defined amygdala activation mask) for faces-shapes contrast	25054	Task fMRI	7373
90th percentile of BOLD effect (in group-defined mask) for shapes activation	25761	Task fMRI	7373
90th percentile of z-statistic (in group-defined mask) for shapes activation	25762	Task fMRI	7373
90th percentile of BOLD effect (in group-defined mask) for faces activation	25763	Task fMRI	7373
90th percentile of z-statistic (in group-defined mask) for faces activation	25764	Task fMRI	7373
90th percentile of BOLD effect (in group-defined mask) for faces-shapes contrast	25765	Task fMRI	7373
90th percentile of BOLD effect (in group-defined amygdala activation mask) for faces-shapes contrast	25767	Task fMRI	7373
90th percentile of z-statistic (in group-defined amygdala activation mask) for faces-shapes contrast	25768	Task fMRI	7373

*A partial correlation matrix with 25 dimensionalities, which had been converted into vectors of length 210 [3,4].

Abbreviations: FLAIR, fluid-attenuated inversion recovery; fMRI, functional magnetic resonance imaging; MRI, magnetic resonance imaging.

Supplementary Fig. S1 Workflow used to model brain age and BAG.

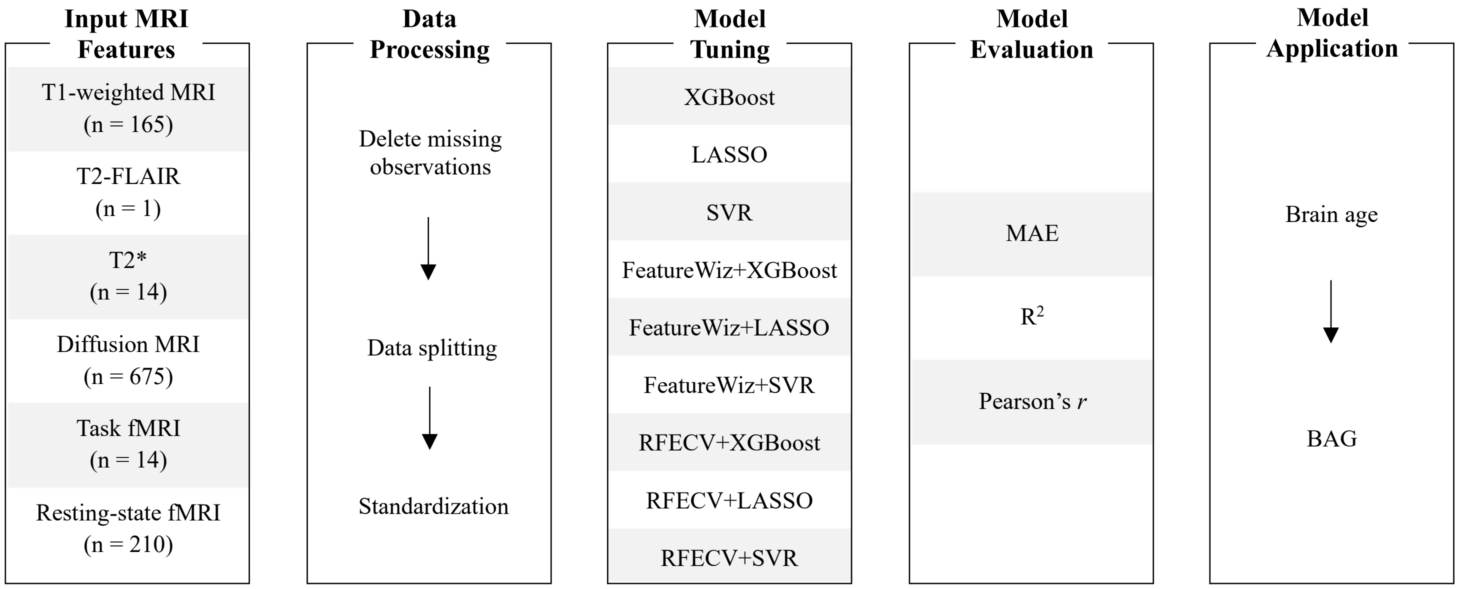

Abbreviations: BAG, brain age gap; fMRI, functional magnetic resonance imaging; LASSO, least absolute shrinkage and selection operator; MAE, mean absolute error; MRI, magnetic resonance imaging; RFECV, Recursive Feature Elimination with Cross Validation; SVR, Support Vector Regression; XGBoost, eXtreme Gradient Boosting.

Supplementary Table S5 Hyperparameter spaces of the nine candidate machine learning models for brain age estimation in Bayesian optimization.

Model	Feature Selector	Hyperparameter	Range
XGBoost	NA	alpha	0.001 to 1000
XGBoost	NA	gamma	0.001 to 1000
XGBoost	NA	learning_rate	0.01 to 0.3
XGBoost	NA	max_depth	2 to 6
XGBoost	NA	n_estimators	200 to 800
XGBoost	NA	reg_lambda	0.001 to 1000
XGBoost	NA	subsample	0.6 to 1
LASSO	NA	alpha	0.07 to 1
SVR	NA	C	1 to 1000
XGBoost	FeatureWiz	alpha	0.001 to 1000
XGBoost	FeatureWiz	gamma	0.001 to 1000
XGBoost	FeatureWiz	learning_rate	0.01 to 0.3
XGBoost	FeatureWiz	max_depth	2 to 6
XGBoost	FeatureWiz	n_estimators	200 to 800
XGBoost	FeatureWiz	reg_lambda	0.001 to 1000
XGBoost	FeatureWiz	subsample	0.6 to 1
LASSO	FeatureWiz	alpha	0.07 to 1
SVR	FeatureWiz	C	1 to 1000
XGBoost	RFECV	alpha	0.001 to 1000
XGBoost	RFECV	gamma	0.001 to 1000
XGBoost	RFECV	learning_rate	0.01 to 0.3
XGBoost	RFECV	max_depth	2 to 6
XGBoost	RFECV	n_estimators	200 to 800
XGBoost	RFECV	reg_lambda	0.001 to 1000
XGBoost	RFECV	subsample	0.6 to 1
LASSO	RFECV	alpha	0.07 to 1
SVR	RFECV	C	1 to 1000

Abbreviations: LASSO, least absolute shrinkage and selection operator; MAE, mean absolute error; NA, not available; RFECV, recursive feature elimination cross-validation; SVR, support vector regression; XGBoost, eXtreme Gradient Boosting.

Supplementary Table S6 Chosen hyperparameters of the nine candidate machine learning models for brain age estimation.

Model	Feature Selector	Hyperparameter	Value
XGBoost	NA	alpha	0.001
XGBoost	NA	gamma	0.010548939
XGBoost	NA	learning_rate	0.086711115
XGBoost	NA	max_depth	3
XGBoost	NA	n_estimators	536
XGBoost	NA	reg_lambda	118.9444064
XGBoost	NA	subsample	0.779595775
LASSO	NA	alpha	0.07
SVR	NA	C	19
XGBoost	FeatureWiz	alpha	24.66941176
XGBoost	FeatureWiz	gamma	2.833421611
XGBoost	FeatureWiz	learning_rate	0.196324798
XGBoost	FeatureWiz	max_depth	2
XGBoost	FeatureWiz	n_estimators	790
XGBoost	FeatureWiz	reg_lambda	1000
XGBoost	FeatureWiz	subsample	0.654883315
LASSO	FeatureWiz	alpha	0.07
SVR	FeatureWiz	C	26
XGBoost	RFECV	alpha	0.539132035
XGBoost	RFECV	gamma	0.001
XGBoost	RFECV	learning_rate	0.067385078
XGBoost	RFECV	max_depth	2
XGBoost	RFECV	n_estimators	732
XGBoost	RFECV	reg_lambda	6.84397712
XGBoost	RFECV	subsample	0.632891091
LASSO	RFECV	alpha	0.07
SVR	RFECV	C	5

Abbreviations: LASSO, least absolute shrinkage and selection operator; MAE, mean absolute error; NA, not available; RFECV, recursive feature elimination cross-validation; SVR, support vector regression; XGBoost, eXtreme Gradient Boosting.

Supplementary Table S7 Performance comparison for nine candidate machine learning models for brain age estimation.

Model	Feature Selector	MAE	R^2	*Pearson's* r	MSE	Explained Variance Score
**Validation set**						
XGBoost	None	3.411	0.650	0.806	18.300	0.651
LASSO	None	**3.232**	**0.690**	**0.830**	16.225	0.692
SVR	None	3.235	0.680	0.825	16.731	0.681
XGBoost	FeatureWiz	3.285	0.676	0.822	16.967	0.677
LASSO	FeatureWiz	3.301	0.677	0.823	16.903	0.679
SVR	FeatureWiz	3.283	0.677	0.823	16.898	0.677
XGBoost	RFECV	3.415	0.650	0.806	18.322	0.651
LASSO	RFECV	3.348	0.666	0.816	17.472	0.668
SVR	RFECV	3.412	0.645	0.803	18.539	0.648
**Testing set**						
XGBoost	None	3.552	0.662	0.814	19.859	0.664
LASSO	None	3.416	0.675	0.822	19.118	0.676
SVR	None	3.455	0.680	0.825	18.811	0.682
XGBoost	FeatureWiz	3.526	0.667	0.817	19.577	0.669
LASSO	FeatureWiz	3.482	0.666	0.816	19.619	0.668
SVR	FeatureWiz	3.491	0.674	0.821	19.178	0.676
XGBoost	RFECV	3.545	0.663	0.814	19.820	0.665
LASSO	RFECV	3.525	0.657	0.811	20.155	0.659
SVR	RFECV	3.548	0.663	0.814	19.834	0.664

Abbreviations: LASSO, least absolute shrinkage and selection operator; MAE, mean absolute error; MSE, mean square error; RFECV, Recursive feature elimination with cross-validation; SVR, Support vector regression; XGBoost, eXtreme Gradient Boosting.

Supplementary Table S8 Coefficients for 285 imaging-derived phenotypes that significantly contributed to brain age estimation in LASSO regression without feature selection.

**Imaging-Derived Phenotype (IDP)**	**Coefficient**
Volume of grey matter (normalized for head size)	-1.3516
Weighted-mean ICVF in tract forceps minor	-0.7169
Mean ISOVF in fornix on FA skeleton	0.6877
Volume of grey matter in Ventral Striatum (left)	-0.6099
Mean FA in superior cerebellar peduncle on FA skeleton (right)	0.5779
Volume of brain stem + 4th ventricle	0.5745
Weighted-mean OD in tract anterior thalamic radiation (right)	-0.5529
Mean L1 in anterior limb of internal capsule on FA skeleton (right)	0.5410
Volume of thalamus (right)	-0.5232
Weighted-mean FA in tract forceps minor	-0.5113
Mean FA in cerebral peduncle on FA skeleton (left)	-0.4964
Volume of grey matter in Putamen (left)	0.4557
Mean L1 in middle cerebellar peduncle on FA skeleton	-0.4240
Volume of grey matter in Insular Cortex (left)	0.4107
Volume of grey matter in VI Cerebellum (left)	-0.4004
Volume of putamen (left)	-0.3795
Median T2star in putamen (left)	-0.3773
Volume of grey matter in IX Cerebellum (left)	0.3740
Mean L1 in anterior limb of internal capsule on FA skeleton (left)	0.3556
Mean MO in fornix cres+stria terminalis on FA skeleton (left)	-0.3367
Weighted-mean OD in tract posterior thalamic radiation (right)	-0.3219
Mean ICVF in superior longitudinal fasciculus on FA skeleton (right)	0.3088
Mean MO in fornix on FA skeleton	0.3075
Weighted-mean ISOVF in tract uncinate fasciculus (left)	0.2999
Volume of grey matter in Frontal Operculum Cortex (right)	-0.2969
Mean L3 in posterior thalamic radiation on FA skeleton (right)	0.2762
Volume of thalamus (left)	-0.2757
Mean L2 in fornix cres+stria terminalis on FA skeleton (left)	0.2661
Mean OD in posterior limb of internal capsule on FA skeleton (right)	0.2650
Mean FA in body of corpus callosum on FA skeleton	0.2633
Weighted-mean MO in tract acoustic radiation (left)	-0.2515
Volume of grey matter in Heschl's Gyrus (includes H1 and H2) (right)	-0.2482
Mean L1 in genu of corpus callosum on FA skeleton	0.2473
Mean L2 in splenium of corpus callosum on FA skeleton	-0.2430
Weighted-mean ISOVF in tract forceps minor	-0.2387
Volume of putamen (right)	-0.2383
Mean OD in anterior limb of internal capsule on FA skeleton (right)	0.2364
Weighted-mean ISOVF in tract superior thalamic radiation (right)	0.2271
Volume of grey matter in Lateral Occipital Cortex, inferior division (left)	0.2193
Mean ICVF in body of corpus callosum on FA skeleton	0.2128
rfMRI partial correlation matrix, dimension 25 (element 180)	0.2090
Mean ICVF in tapetum on FA skeleton (right)	-0.2087
Median z-statistic (in group-defined amygdala activation mask) for faces-shapes contrast	-0.2082
Mean OD in pontine crossing tract on FA skeleton	0.2078
Mean L3 in retrolenticular part of internal capsule on FA skeleton (left)	-0.2076
Weighted-mean ISOVF in tract superior longitudinal fasciculus (right)	0.2068
Mean ISOVF in superior corona radiata on FA skeleton (right)	0.1990
rfMRI partial correlation matrix, dimension 25 (element 127)	0.1965
Mean L1 in sagittal stratum on FA skeleton (right)	-0.1930
Mean ICVF in fornix cres+stria terminalis on FA skeleton (right)	-0.1921
Weighted-mean L3 in tract medial lemniscus (right)	-0.1885
rfMRI partial correlation matrix, dimension 25 (element 44)	-0.1877
Volume of grey matter in Inferior Temporal Gyrus, temporooccipital part (left)	0.1851
Mean ICVF in retrolenticular part of internal capsule on FA skeleton (right)	0.1833

Mean FA in superior cerebellar peduncle on FA skeleton (left)	0.1828
rfMRI partial correlation matrix, dimension 25 (element 172)	0.1826
Weighted-mean L1 in tract parahippocampal part of cingulum (left)	-0.1823
Weighted-mean ICVF in tract superior longitudinal fasciculus (right)	0.1801
Mean OD in superior cerebellar peduncle on FA skeleton (left)	-0.1790
Volume of grey matter in Frontal Orbital Cortex (left)	-0.1696
Volume of grey matter in X Cerebellum (left)	-0.1652
Volume of grey matter in Paracingulate Gyrus (right)	-0.1651
Weighted-mean L1 in tract corticospinal tract (left)	-0.1642
Mean ICVF in medial lemniscus on FA skeleton (left)	0.1599
rfMRI partial correlation matrix, dimension 25 (element 22)	0.1593
Volume of grey matter in Hippocampus (left)	-0.1592
Weighted-mean FA in tract middle cerebellar peduncle	-0.1569
Mean ICVF in corticospinal tract on FA skeleton (right)	0.1564
90th percentile of BOLD effect (in group-defined mask) for faces-shapes contrast	-0.1537
Volume of grey matter in Crus II Cerebellum (vermis)	0.1511
rfMRI partial correlation matrix, dimension 25 (element 96)	0.1498
Weighted-mean ICVF in tract middle cerebellar peduncle	-0.1496
Weighted-mean OD in tract anterior thalamic radiation (left)	-0.1485
Mean L1 in superior corona radiata on FA skeleton (left)	0.1481
rfMRI partial correlation matrix, dimension 25 (element 151)	-0.1473
rfMRI partial correlation matrix, dimension 25 (element 131)	-0.1440
Mean L1 in superior longitudinal fasciculus on FA skeleton (left)	-0.1422
Mean ISOVF in cingulum cingulate gyrus on FA skeleton (right)	-0.1402
rfMRI partial correlation matrix, dimension 25 (element 89)	0.1397
Mean ICVF in corticospinal tract on FA skeleton (left)	0.1388
Volume of grey matter in Frontal Operculum Cortex (left)	-0.1377
rfMRI partial correlation matrix, dimension 25 (element 46)	-0.1336
Volume of grey matter in X Cerebellum (right)	-0.1322
Mean FA in fornix on FA skeleton	-0.1321
Median T2star in thalamus (left)	0.1312
Mean FA in corticospinal tract on FA skeleton (right)	0.1299
Weighted-mean MO in tract acoustic radiation (right)	-0.1284
Volume of grey matter in Lingual Gyrus (right)	-0.1282
Mean L1 in cerebral peduncle on FA skeleton (right)	-0.1270
rfMRI partial correlation matrix, dimension 25 (element 146)	0.1266
rfMRI partial correlation matrix, dimension 25 (element 135)	-0.1256
Weighted-mean L1 in tract corticospinal tract (right)	-0.1253
Volume of grey matter in Angular Gyrus (left)	0.1231
Mean L2 in cingulum cingulate gyrus on FA skeleton (right)	-0.1207
Volume of grey matter in Supramarginal Gyrus, anterior division (right)	0.1195
Volume of grey matter in Crus I Cerebellum (vermis)	-0.1187
Volume of grey matter in Planum Polare (left)	-0.1185
Volume of grey matter in Pallidum (left)	0.1175
Volume of grey matter in Planum Polare (right)	-0.1166
Weighted-mean MO in tract forceps major	-0.1160
Mean ICVF in posterior corona radiata on FA skeleton (left)	0.1153
Weighted-mean ICVF in tract anterior thalamic radiation (right)	-0.1150
Volume of grey matter in Frontal Orbital Cortex (right)	-0.1143
rfMRI partial correlation matrix, dimension 25 (element 159)	0.1122
rfMRI partial correlation matrix, dimension 25 (element 161)	-0.1119
Weighted-mean OD in tract superior longitudinal fasciculus (left)	0.1116
Weighted-mean L2 in tract posterior thalamic radiation (right)	0.1114
rfMRI partial correlation matrix, dimension 25 (element 49)	-0.1111
Weighted-mean ICVF in tract corticospinal tract (right)	0.1107
rfMRI partial correlation matrix, dimension 25 (element 202)	0.1087
Weighted-mean L1 in tract acoustic radiation (left)	-0.1083
Mean MD in cingulum cingulate gyrus on FA skeleton (right)	-0.1082
Volume of grey matter in Frontal Medial Cortex (right)	-0.1079
Mean L1 in superior corona radiata on FA skeleton (right)	0.1078

rfMRI partial correlation matrix, dimension 25 (element 124)	-0.1065
Mean FA in middle cerebellar peduncle on FA skeleton	-0.1061
Volume of grey matter in Occipital Fusiform Gyrus (left)	0.1054
90th percentile of z-statistic (in group-defined amygdala activation mask) for faces-shapes contrast	-0.1052
rfMRI partial correlation matrix, dimension 25 (element 58)	0.1025
Mean ISOVF in inferior cerebellar peduncle on FA skeleton (left)	-0.1009
Mean MO in medial lemniscus on FA skeleton (right)	0.1004
Mean FA in fornix cres+stria terminalis on FA skeleton (right)	-0.0995
Volume of grey matter in Thalamus (right)	0.0991
Mean ICVF in posterior thalamic radiation on FA skeleton (left)	-0.0989
Weighted-mean OD in tract posterior thalamic radiation (left)	-0.0978
rfMRI partial correlation matrix, dimension 25 (element 66)	-0.0954
rfMRI partial correlation matrix, dimension 25 (element 60)	-0.0949
Mean OD in superior cerebellar peduncle on FA skeleton (right)	-0.0947
Mean MO in medial lemniscus on FA skeleton (left)	0.0945
rfMRI partial correlation matrix, dimension 25 (element 155)	0.0934
rfMRI partial correlation matrix, dimension 25 (element 144)	-0.0924
Weighted-mean MO in tract cingulate gyrus part of cingulum (left)	-0.0910
Volume of grey matter in Inferior Temporal Gyrus, posterior division (right)	-0.0895
rfMRI partial correlation matrix, dimension 25 (element 156)	0.0885
Weighted-mean MD in tract corticospinal tract (right)	-0.0885
rfMRI partial correlation matrix, dimension 25 (element 130)	-0.0881
Volume of grey matter in Crus II Cerebellum (right)	-0.0856
Volume of grey matter in VIIIb Cerebellum (vermis)	0.0850
Median T2star in thalamus (right)	0.0843
Volume of grey matter in Supramarginal Gyrus, posterior division (right)	-0.0840
rfMRI partial correlation matrix, dimension 25 (element 103)	-0.0840
Mean MD in uncinate fasciculus on FA skeleton (right)	0.0835
Mean ICVF in superior fronto-occipital fasciculus on FA skeleton (right)	-0.0832
rfMRI partial correlation matrix, dimension 25 (element 204)	0.0819
Weighted-mean ISOVF in tract middle cerebellar peduncle	-0.0816
Mean MO in fornix cres+stria terminalis on FA skeleton (right)	-0.0807
Mean L1 in superior fronto-occipital fasciculus on FA skeleton (left)	0.0799
Mean MO in external capsule on FA skeleton (right)	-0.0797
Mean OD in fornix on FA skeleton	-0.0796
rfMRI partial correlation matrix, dimension 25 (element 25)	-0.0790
rfMRI partial correlation matrix, dimension 25 (element 121)	-0.0781
Weighted-mean ISOVF in tract parahippocampal part of cingulum (right)	0.0780
rfMRI partial correlation matrix, dimension 25 (element 97)	0.0779
rfMRI partial correlation matrix, dimension 25 (element 125)	0.0772
Mean ICVF in inferior cerebellar peduncle on FA skeleton (left)	-0.0749
Volume of grey matter in Planum Temporale (left)	-0.0745
Mean FA in inferior cerebellar peduncle on FA skeleton (left)	-0.0728
Weighted-mean MO in tract posterior thalamic radiation (left)	-0.0724
Mean OD in posterior thalamic radiation on FA skeleton (right)	0.0715
rfMRI partial correlation matrix, dimension 25 (element 84)	-0.0707
rfMRI partial correlation matrix, dimension 25 (element 55)	0.0682
rfMRI partial correlation matrix, dimension 25 (element 142)	-0.0678
90th percentile of BOLD effect (in group-defined amygdala activation mask) for faces-shapes contrast	0.0666
Mean OD in posterior limb of internal capsule on FA skeleton (left)	0.0662
Mean FA in anterior limb of internal capsule on FA skeleton (left)	0.0661
rfMRI partial correlation matrix, dimension 25 (element 101)	0.0657
rfMRI partial correlation matrix, dimension 25 (element 31)	0.0647
rfMRI partial correlation matrix, dimension 25 (element 205)	0.0644
rfMRI partial correlation matrix, dimension 25 (element 35)	-0.0634
Weighted-mean L1 in tract superior thalamic radiation (right)	0.0622
Mean MD in retrolenticular part of internal capsule on FA skeleton (right)	-0.0617
Weighted-mean MO in tract medial lemniscus (left)	-0.0614

Mean OD in external capsule on FA skeleton (right)	0.0612
Mean ISOVF in posterior limb of internal capsule on FA skeleton (left)	-0.0589
Median T2star in accumbens (left)	-0.0570
Weighted-mean ISOVF in tract anterior thalamic radiation (left)	-0.0569
rfMRI partial correlation matrix, dimension 25 (element 169)	0.0560
Mean MO in inferior cerebellar peduncle on FA skeleton (right)	-0.0556
rfMRI partial correlation matrix, dimension 25 (element 92)	-0.0552
Mean OD in cingulum cingulate gyrus on FA skeleton (left)	-0.0534
rfMRI partial correlation matrix, dimension 25 (element 94)	0.0528
Mean OD in uncinate fasciculus on FA skeleton (right)	0.0502
Volume of grey matter in Cingulate Gyrus, anterior division (left)	-0.0490
Weighted-mean OD in tract uncinate fasciculus (right)	-0.0490
Weighted-mean OD in tract parahippocampal part of cingulum (right)	0.0468
Mean OD in anterior corona radiata on FA skeleton (right)	-0.0465
Weighted-mean ISOVF in tract posterior thalamic radiation (right)	0.0450
rfMRI partial correlation matrix, dimension 25 (element 21)	-0.0448
rfMRI partial correlation matrix, dimension 25 (element 194)	0.0446
Volume of grey matter in VI Cerebellum (right)	-0.0435
rfMRI partial correlation matrix, dimension 25 (element 128)	0.0433
Mean ISOVF in cingulum hippocampus on FA skeleton (left)	0.0425
Mean OD in corticospinal tract on FA skeleton (left)	-0.0416
rfMRI partial correlation matrix, dimension 25 (element 143)	0.0412
Mean MD in cingulum hippocampus on FA skeleton (right)	-0.0410
rfMRI partial correlation matrix, dimension 25 (element 39)	-0.0407
Volume of amygdala (left)	0.0403
rfMRI partial correlation matrix, dimension 25 (element 51)	-0.0399
Mean L2 in posterior corona radiata on FA skeleton (left)	-0.0396
rfMRI partial correlation matrix, dimension 25 (element 210)	0.0390
rfMRI partial correlation matrix, dimension 25 (element 95)	-0.0380
Mean L2 in fornix cres+stria terminalis on FA skeleton (right)	0.0377
rfMRI partial correlation matrix, dimension 25 (element 50)	-0.0375
rfMRI partial correlation matrix, dimension 25 (element 15)	0.0372
Weighted-mean MO in tract superior longitudinal fasciculus (right)	-0.0366
Weighted-mean MD in tract superior thalamic radiation (left)	0.0364
Mean OD in cerebral peduncle on FA skeleton (right)	0.0360
Mean ISOVF in anterior limb of internal capsule on FA skeleton (right)	0.0348
Volume of grey matter in Subcallosal Cortex (right)	0.0343
Volume of grey matter in VIIb Cerebellum (vermis)	-0.0337
Volume of grey matter in Occipital Pole (left)	-0.0329
rfMRI partial correlation matrix, dimension 25 (element 189)	0.0328
Median T2star in pallidum (right)	0.0326
rfMRI partial correlation matrix, dimension 25 (element 183)	0.0325
Mean MD in cingulum cingulate gyrus on FA skeleton (left)	-0.0322
Mean L1 in pontine crossing tract on FA skeleton	-0.0321
rfMRI partial correlation matrix, dimension 25 (element 140)	-0.0305
rfMRI partial correlation matrix, dimension 25 (element 75)	0.0302
rfMRI partial correlation matrix, dimension 25 (element 5)	0.0301
Volume of grey matter in Brain-Stem	-0.0301
rfMRI partial correlation matrix, dimension 25 (element 176)	0.0294
rfMRI partial correlation matrix, dimension 25 (element 85)	-0.0294
Mean L3 in posterior limb of internal capsule on FA skeleton (left)	-0.0290
rfMRI partial correlation matrix, dimension 25 (element 82)	-0.0284
rfMRI partial correlation matrix, dimension 25 (element 123)	-0.0283
90th percentile of BOLD effect (in group-defined mask) for shapes activation	0.0279
Volume of grey matter in Lingual Gyrus (left)	-0.0278
rfMRI partial correlation matrix, dimension 25 (element 26)	0.0269
Mean ICVF in superior cerebellar peduncle on FA skeleton (right)	0.0266
Weighted-mean OD in tract inferior longitudinal fasciculus (right)	0.0264
rfMRI partial correlation matrix, dimension 25 (element 141)	0.0259
rfMRI partial correlation matrix, dimension 25 (element 106)	-0.0257

rfMRI partial correlation matrix, dimension 25 (element 16)	0.0245
rfMRI partial correlation matrix, dimension 25 (element 112)	0.0243
Volume of grey matter in I-IV Cerebellum (right)	-0.0236
rfMRI partial correlation matrix, dimension 25 (element 116)	-0.0235
Weighted-mean ISOVF in tract anterior thalamic radiation (right)	-0.0232
Median BOLD effect (in group-defined mask) for faces activation	0.0229
rfMRI partial correlation matrix, dimension 25 (element 64)	-0.0224
Volume of grey matter in IX Cerebellum (right)	0.0214
Volume of grey matter in Temporal Fusiform Cortex, posterior division (left)	-0.0212
rfMRI partial correlation matrix, dimension 25 (element 196)	-0.0201
Weighted-mean MO in tract posterior thalamic radiation (right)	-0.0199
rfMRI partial correlation matrix, dimension 25 (element 29)	0.0190
rfMRI partial correlation matrix, dimension 25 (element 71)	0.0189
Mean L2 in posterior limb of internal capsule on FA skeleton (right)	0.0187
rfMRI partial correlation matrix, dimension 25 (element 56)	-0.0180
rfMRI partial correlation matrix, dimension 25 (element 17)	-0.0175
Volume of grey matter in Inferior Temporal Gyrus, anterior division (right)	-0.0168
Volume of grey matter in Juxtapositional Lobule Cortex (formerly Supplementary Motor Cortex) (left)	-0.0155
Mean L2 in sagittal stratum on FA skeleton (left)	-0.0150
rfMRI partial correlation matrix, dimension 25 (element 164)	0.0149
rfMRI partial correlation matrix, dimension 25 (element 133)	0.0144
Volume of grey matter in Lateral Occipital Cortex, superior division (right)	-0.0140
rfMRI partial correlation matrix, dimension 25 (element 107)	0.0136
Volume of grey matter in Cuneal Cortex (right)	0.0131
Mean L1 in cerebral peduncle on FA skeleton (left)	-0.0126
Volume of grey matter in Superior Temporal Gyrus, posterior division (left)	-0.0125
Volume of grey matter in Supramarginal Gyrus, anterior division (left)	0.0123
rfMRI partial correlation matrix, dimension 25 (element 113)	-0.0123
Mean L2 in retrolenticular part of internal capsule on FA skeleton (right)	-0.0121
rfMRI partial correlation matrix, dimension 25 (element 80)	0.0118
Mean OD in retrolenticular part of internal capsule on FA skeleton (left)	0.0109
Mean MO in cerebral peduncle on FA skeleton (left)	-0.0108
Volume of grey matter in Superior Parietal Lobule (left)	-0.0106
rfMRI partial correlation matrix, dimension 25 (element 98)	0.0094
Weighted-mean OD in tract cingulate gyrus part of cingulum (left)	0.0089
Mean L1 in uncinate fasciculus on FA skeleton (right)	0.0084
rfMRI partial correlation matrix, dimension 25 (element 7)	-0.0081
Weighted-mean OD in tract uncinate fasciculus (left)	0.0079
rfMRI partial correlation matrix, dimension 25 (element 197)	-0.0077
Median T2star in pallidum (left)	0.0067
rfMRI partial correlation matrix, dimension 25 (element 190)	0.0066
rfMRI partial correlation matrix, dimension 25 (element 206)	0.0063
Weighted-mean MO in tract forceps minor	-0.0062
Volume of grey matter in Frontal Pole (left)	-0.0060
Mean L3 in posterior thalamic radiation on FA skeleton (left)	0.0059
rfMRI partial correlation matrix, dimension 25 (element 136)	-0.0057
rfMRI partial correlation matrix, dimension 25 (element 179)	0.0053
Mean MO in cingulum hippocampus on FA skeleton (right)	0.0044
Mean OD in superior fronto-occipital fasciculus on FA skeleton (left)	-0.0034
Weighted-mean OD in tract acoustic radiation (left)	0.0023
rfMRI partial correlation matrix, dimension 25 (element 186)	0.0019
Volume of grey matter in VI Cerebellum (vermis)	-0.0013
rfMRI partial correlation matrix, dimension 25 (element 87)	-0.0008

Supplementary Fig. S2 Original and corrected brain age as a function of chronological age in the (A) testing set and (B) validation set.

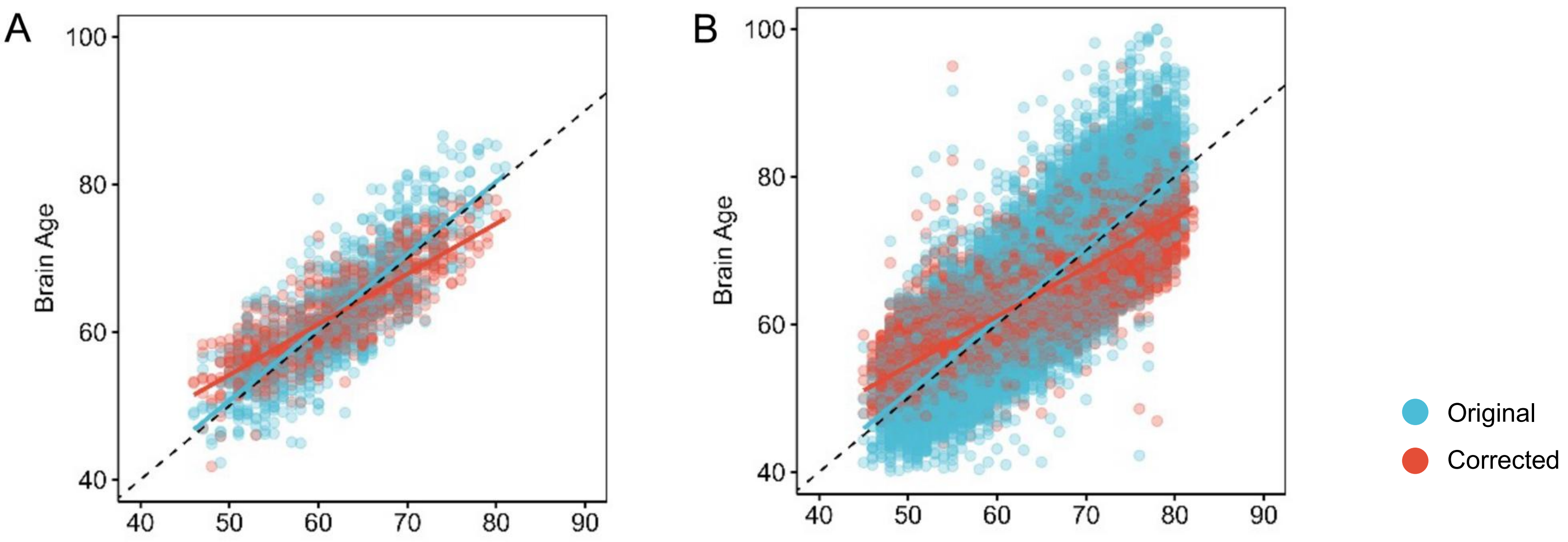

Supplementary Table S9 Baseline characteristics of the entire UK Biobank, UK Biobank neuroimaging cohort, and current study sample.

Characteristic	UK Biobank	UK Biobank neuroimaging cohort	Current study sample
N	502,366	42,806	21,473
Age (y)	56.5 ± 8.09	55.0 ± 7.5	54.9 ± 7.5
Sex			
Male	229,066 (45.6)	20,220 (47.2)	9,965 (46.4)
Female	273,300 (54.4)	22,586 (52.8)	11,508 (53.6)
Race			
White	454,133 (90.4)	39,501 (92.3)	19,910 (92.7)
Other	45,457 (9.0)	3,191 (7.5)	1,505 (7.0)
College/university education			
Yes	162,523 (32.4)	20,067 (46.9)	10,816 (50.4)
No	333,453 (66.4)	22,598 (52.8)	10,610 (49.4)
Socioeconomic status	-1.3 ± 3.1	-1.9 ± 2.7	-1.9 ± 2.7
Smoking status			
Never	273,449 (54.4)	25,981 (60.7)	13,163 (61.3)
Former	173,008 (34.4)	14,073 (32.9)	7,044 (32.8)
Current	52,961 (10.5)	2,655 (6.2)	1,218 (5.7)
Physical activity			
Low	76,190 (15.2)	6,675 (15.6)	3,378 (15.7)
Moderate	163,987 (32.6)	15,340 (35.8)	7,943 (37.0)
High	162,096 (32.3)	14,442 (33.7)	7,461 (34.7)
Body mass index, kg/m^2			
<18.5	2,626 (0.5)	188 (0.4)	90 (0.4)
18.5 to <25	162,352 (32.3)	16,708 (39.0)	8,837 (41.2)
25 to <30	212,062 (42.2)	18,258 (42.7)	9,052 (42.2)
≥30	122,222 (24.3)	7,590 (17.7)	3,473 (16.2)
Hypertension	143,663 (28.6)	9,320 (21.8)	4,383 (20.4)
Cardiovascular disease	34,192 (6.8)	1,703 (4.0)	771 (3.6)
Type 2 diabetes	37,160 (7.4)	1,646 (3.8)	766 (3.6)

Missing data: race = 2,776; education = 6,390; socioeconomic status = 623; smoking status = 2,948; physical activity = 100,093; body mass index = 3,104; hypertension = 796; cardiovascular disease = 2,030; type 2 diabetes = 622.

Supplementary Table S10 Comparison of baseline characteristics between the study sample and excluded participants.

Characteristic	UK Biobank	Excluded	Included	*p*-value
N	502,366	480,893	21,473	
Age (y)	56.5 ± 8.09	56.6 ± 8.1	54.9 ± 7.5	<0.001
Sex				0.01
Male	229,066 (45.6)	219,101 (45.6)	9,965 (46.4)	
Female	273,300 (54.4)	261,792 (54.4)	11,508 (53.6)	
Race				<0.001
White	454,133 (90.4)	434,223 (90.3)	19,910 (92.7)	
Other	45,457 (9.0)	43,952 (9.1)	1,505 (7.0)	
College/university education				<0.001
Yes	162,523 (32.4)	151,707 (31.5)	10,816 (50.4)	
No	333,453 (66.4)	322,843 (67.1)	10,610 (49.4)	
Socioeconomic status	-1.3 ± 3.1	-1.3 ± 3.1	-1.9 ± 2.7	<0.001
Smoking status				
Never	273,449 (54.4)	260,286 (54.1)	13,163 (61.3)	<0.001
Former	173,008 (34.4)	165,964 (34.5)	7,044 (32.8)	
Current	52,961 (10.5)	51,743 (10.8)	1,218 (5.7)	
Physical activity				<0.001
Low	76,190 (15.2)	72,812 (15.1)	3,378 (15.7)	
Moderate	163,987 (32.6)	156,044 (32.4)	7,943 (37.0)	
High	162,096 (32.3)	154,635 (32.2)	7,461 (34.7)	
Body mass index, kg/m^2				<0.001
<18.5	2,626 (0.5)	2,536 (0.5)	90 (0.4)	
18.5 to <25	162,352 (32.3)	153,515 (31.9)	8,837 (41.2)	
25 to <30	212,062 (42.2)	203,010 (42.2)	9,052 (42.2)	
≥30	122,222 (24.3)	118,749 (24.7)	3,473 (16.2)	
Hypertension	143,663 (28.6)	139,280 (28.9)	4,383 (20.4)	<0.001
Cardiovascular disease	34,192 (6.8)	33,421 (6.9)	771 (3.6)	<0.001
Type 2 diabetes	37,160 (7.4)	36,394 (7.6)	766 (3.6)	<0.001

Missing data: race = 2,776; education = 6,390; socioeconomic status = 623; smoking status = 2,948; physical activity = 100,093; body mass index = 3,104; hypertension = 796; cardiovascular disease = 2,030; type 2 diabetes = 622.

Supplementary Table S11 Number of available dietary assessments.

Dietary assessment	N (%)
Baseline, 2009-2010	5,055 (23.5)
Cycle 1, Feb. 2011-April 2011	11,698 (54.5)
Cycle 2, June 2011-Sept. 2011	10,035 (46.7)
Cycle 3, Oct. 2011-Dec. 2011	12,775 (59.5)
Cycle 4, April 2012-June 2012	12,440 (57.9)
**Total number of assessments**	**N (%)**
1	6,244 (29.1)
2	5,285 (24.6)
3	5,257 (24.5)
4	4,017 (18.7)
5	670 (3.1)

Supplementary Fig. S3 Bland-Altman plots comparing Dietary Inflammatory Index (DII) scores (A) from baseline and visit 5 assessments, and (B) from participants' first and last assessments.

A.

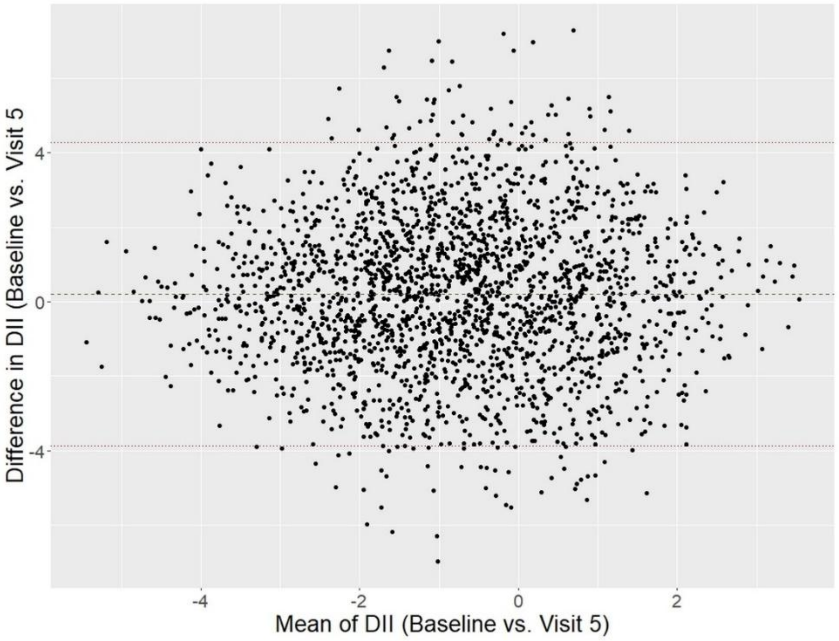

B.

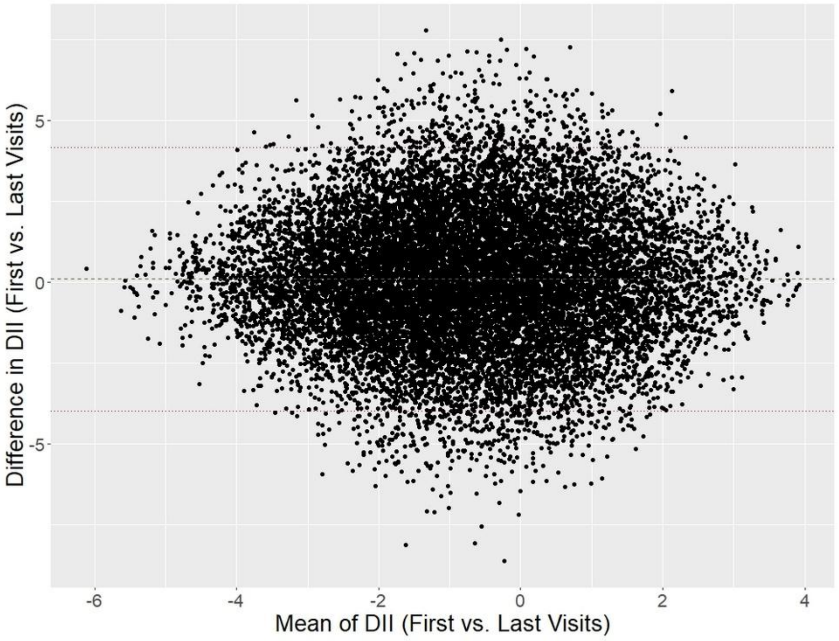

Abbreviations: DII, Dietary Inflammatory Index.

Supplementary Table S12 Interactions of DII with age, PRS_{AD}, and *APOE*4 in relation to BAG.

Interaction	BAG, $\hat{\beta}$ (95% CI)	*p*-value
**DII*Age group (ref. = middle-aged)**		
Continuous DII	0.06 (-0.02, 0.14)	0.17
Groups (ref. = Group 1)		
Group 2, older-aged	0.27 (-0.14, 0.68)	0.20
Group 3, older-aged	0.32 (-0.11, 0.74)	0.15
Group 4, older-aged	0.41 (-0.17, 0.98)	0.17
**DII*PRS_{AD} (ref. = low PRS_{AD})**		
Continuous DII, moderate PRS_{AD}	-0.03 (-0.12, 0.06)	0.48
Continuous DII, high PRS_{AD}	-0.03 (-0.12, 0.06)	0.51
Groups (ref. = Group 1)		
Group 2, moderate PRS_{AD}	-0.04 (-0.52, 0.44)	0.87
Group 3, moderate PRS_{AD}	-0.13 (-0.63, 0.36)	0.60
Group 4, moderate PRS_{AD}	-0.14 (-0.78, 0.49)	0.66
Group 2, high PRS_{AD}	-0.01 (-0.49, 0.46)	0.96
Group 3, high PRS_{AD}	0.06 (-0.44, 0.55)	0.83
Group 4, high PRS_{AD}	-0.25 (-0.89, 0.39)	0.44
**DII*_APOE_4 status (ref. = non-carriers)**		
Continuous DII	-0.05 (-0.14, 0.03)	0.23
Groups (ref. = Group 1)		
Group 2, *APOE*4 carriers	0.11 (-0.36, 0.58)	0.66
Group 3, *APOE*4 carriers	0.02 (-0.46, 0.51)	0.93
Group 4, *APOE*4 carriers	-0.19 (-0.81, 0.44)	0.55

Models were adjusted for age (except for DII*age interaction models), sex, race, education, socioeconomic status, energy intake, body mass index, smoking status, physical activity, cardiovascular disease, type 2 diabetes, hypertension, and Alzheimer's disease polygenic risk score (except for DII*PRS_{AD} and DII*_APOE_ interaction models).

Abbreviations: *APOE*4, Apolipoprotein E ε4 allele; BAG, brain age gap; CI, confidence interval; DII, Dietary Inflammatory Index; PRS_{AD}, Alzheimer's disease polygenic risk score; ref., reference.

Supplementary Table S13 The association of DII with BAG stratified by age, PRS_{AD}, and *APOE*4 status.

DII	N	BAG, $\widehat{\beta}$ (95% CI)	*p*-value
Age groups			
**Middle-aged (40-59 years)**	14,557		
Continuous DII	14,557	0.08 (0.02, 0.13)	0.005
Groups			
Group 1	3,041	Reference	
Group 2	5,098	-0.01 (-0.25, 0.23)	0.92
Group 3	4,470	0.22 (-0.05, 0.48)	0.11
Group 4	1,948	0.49 (0.14, 0.83)	0.005
**Older-aged (≥60 years)**	6,916		
Continuous DII	6,916	0.13 (0.04, 0.22)	0.003
Groups			
Group 1	1,863	Reference	
Group 2	2,474	0.27 (-0.10, 0.64)	0.15
Group 3	1,916	0.54 (0.12, 0.96)	0.01
Group 4	663	0.87 (0.28, 1.47)	0.004
PRS_{AD}			
**Low risk**	7,032		
Continuous DII	7,032	0.06 (-0.02, 0.14)	0.11
Groups			
Group 1	1,564	Reference	
Group 2	2,501	0.01 (-0.34, 0.37)	0.93
Group 3	2,119	0.17 (-0.22, 0.57)	0.39
Group 4	848	0.43 (-0.10, 0.95)	0.11
**Moderate risk**	7,031		
Continuous DII	7,031	0.09 (0.01, 0.17)	0.03
Groups			
Group 1	1,593	Reference	
Group 2	2,437	0.09 (-0.27, 0.44)	0.62
Group 3	2,111	0.27 (-0.13, 0.66)	0.19
Group 4	890	0.65 (0.13, 1.18)	0.01
**High risk**	7,032		
Continuous DII	7,032	0.07 (-0.01, 0.14)	0.08
Groups			
Group 1	1,673	Reference	
Group 2	2,497	0.05 (-0.29, 0.39)	0.78
Group 3	2,035	0.35 (-0.03, 0.74)	0.07
Group 4	827	0.41 (-0.09, 0.92)	0.11
***APOE*4 status**			
***APOE*4 non-carriers**	13,169		
Continuous DII	13,169	0.09 (0.04, 0.15)	0.001
Groups			
Group 1	2,993	Reference	
Group 2	4,657	0.01 (-0.24, 0.26)	0.93
Group 3	3,906	0.27 (-0.02, 0.55)	0.06
Group 4	1,613	0.48 (0.10, 0.86)	0.01
***APOE*4 carriers**	4,977		
Continuous DII	4,977	0.04 (-0.05, 0.13)	0.40
Groups			
Group 1	1,202	Reference	
Group 2	1,725	0.12 (-0.30, 0.54)	0.58

Group 3	1,451	0.33 (-0.15, 0.80)	0.18
Group 4	599	0.38 (-0.24, 1.01)	0.23

Models were adjusted for age (except for age-stratified models), sex, race, education, socioeconomic status, energy intake, body mass index, smoking status, physical activity, cardiovascular disease, type 2 diabetes, hypertension, and Alzheimer's disease polygenic risk score (except for PRS_{AD} -stratified models).

Abbreviations: *APOE*4, Apolipoprotein E ε4 allele; BAG, brain age gap; CI, confidence interval; DII, Dietary Inflammatory Index; PRS_{AD}, Alzheimer's disease polygenic risk score.

Supplementary Table S14 Associations between baseline DII and INFLA-score.

DII	N	INFLA-score<br>$\hat{\beta}$ (95% CI)	*p*-value
Continuous	4,439	0.18 (0.06, 0.29)	0.002
Groups			
Group 1	1,341	Reference	
Group 2	1,615	0.37 (-0.07, 0.82)	0.10
Group 3	1,167	0.40 (-0.13, 0.92)	0.14
Group 4	316	1.22 (0.40, 2.05)	0.004

Models were adjusted for age, sex, race, education, socioeconomic status, energy intake, body mass index, smoking status, physical activity, cardiovascular disease, type 2 diabetes, hypertension, and Alzheimer’s disease polygenic risk score.

Abbreviations: DII, Dietary Inflammatory Index; INFLA-score, inflammation score.

Supplementary Table S15 The association of DII with BAG using imputed data for missing covariates.

DII	BAG, $\hat{\beta}$ (95% CI)	*p*-value
**Continuous**	0.07 (0.03, 0.12)	0.002
**Groups**		
Group 1 (< -2)	Reference	
Group 2 (-2 to < 0)	0.05 (-0.15, 0.25)	0.60
Group 3 (0 to < 2)	0.26 (0.03, 0.48)	0.02
Group 4 (≥ 2)	0.50 (0.20, 0.80)	0.001

Models were adjusted for age, sex, race, education, socioeconomic status, energy intake, body mass index, smoking status, physical activity, cardiovascular disease, type 2 diabetes, hypertension, and Alzheimer's disease polygenic risk score.

Abbreviations: BAG, brain age gap; CI, confidence interval; DII, Dietary Inflammatory Index; PRS_{AD}, Alzheimer's disease polygenic risk score; ref., reference.

Supplementary Table S16 The association of DII with BAG according to number and quality of dietary assessments.

DII	N	BAG, $\widehat{\beta}$ (95% CI)	*p*-value
**1 dietary assessment (n=6,244)**			
**Continuous**	6,244	0.08 (0.002, 0.15)	0.04
**Groups**			
Group 1 (< -2)	1,389	Reference	
Group 2 (-2 to < 0)	1,898	-0.16 (-0.54, 0.22)	0.41
Group 3 (0 to < 2)	1,815	0.23 (-0.18, 0.64)	0.28
Group 4 (≥ 2)	1,142	0.40 (-0.10, 0.90)	0.12
**≥2 dietary assessments (n=15,229)**			
**Continuous**	15,229	0.07 (0.01, 0.12)	0.02
**Groups**			
Group 1 (< -2)	3,515	Reference	
Group 2 (-2 to < 0)	5,674	0.13 (-0.11, 0.37)	0.28
Group 3 (0 to < 2)	4,571	0.27 (0.003, 0.54)	0.048
Group 4 (≥ 2)	1,469	0.50 (0.12, 0.88)	0.01
**≥4 dietary assessments (n=4,687)**			
**Continuous**	4,687	0.13 (0.02, 0.24)	0.02
**Groups**			
Group 1 (< -2)	1,067	Reference	
Group 2 (-2 to < 0)	1,850	0.31 (-0.11, 0.74)	0.15
Group 3 (0 to < 2)	1,402	0.70 (0.21, 1.19)	0.005
Group 4 (≥ 2)	368	0.63 (-0.11, 1.38)	0.09
**Typical dietary intake (n=13,201)**			
Continuous	13,201	0.09 (0.04, 0.15)	0.001
Groups			
Group 1 (< -2)	3,394	Reference	
Group 2 (-2 to < 0)	4,624	0.06 (-0.18, 0.31)	0.62
Group 3 (0 to < 2)	3,741	0.36 (0.08, 0.64)	0.01
Group 4 (≥ 2)	1,442	0.67 (0.28, 1.05)	0.0006
**≥2 assessments of typical dietary intake (n=8,491)**			
Continuous	8,491	0.07 (-0.002, 0.14)	0.06
Groups			
Group 1 (< -2)	2,280	Reference	
Group 2 (-2 to < 0)	3,151	0.17 (-0.14, 0.47)	0.28
Group 3 (0 to < 2)	2,376	0.34 (-0.01, 0.69)	0.06
Group 4 (≥ 2)	684	0.59 (0.08, 1.11)	0.02

Models were adjusted for age, sex, race, education, socioeconomic status, energy intake, body mass index, smoking status, physical activity, cardiovascular disease, type 2 diabetes, hypertension, and Alzheimer's disease polygenic risk score.

Abbreviations: BAG, brain age gap; CI, confidence interval; DII, Dietary Inflammatory Index.

Supplementary Table S17 The association of DII with BAG adjusted for *APOE*4 status.

DII	BAG, $\widehat{\beta}$ (95% CI)	*p*-value
**Continuous**	0.08 (0.03, 0.13)	0.001
**Groups**		
Group 1 (< -2)	Reference	
Group 2 (-2 to < 0)	0.04 (-0.17, 0.26)	0.70
Group 3 (0 to < 2)	0.28 (0.04, 0.52)	0.02
Group 4 (≥ 2)	0.46 (0.14, 0.78)	0.005

Models were adjusted for age, sex, race, education, socioeconomic status, energy intake, body mass index, smoking status, physical activity, cardiovascular disease, type 2 diabetes, hypertension, and *APOE*4 status.

Supplementary Table S18 Interactions of DII with sex, body mass index, waist circumference, and cardiometabolic diseases in relation to BAG.

Interaction	BAG, $\hat{\beta}$ (95% CI)	*p*-value
**DII*Sex (ref. = female)**		
Continuous DII	0.07 (-0.004, 0.14)	0.06
Groups (ref. = Group 1)		
Group 2, male	0.22 (-0.17, 0.61)	0.28
Group 3, male	0.46 (0.05, 0.87)	0.03
Group 4, male	0.39 (-0.13, 0.92)	0.14
**DII*BMI (ref. = normal)**		
Continuous DII, underweight	0.12 (-0.44, 0.68)	0.68
Continuous DII, overweight	-0.01 (-0.09, 0.07)	0.79
Continuous DII, obese	-0.04 (-0.14, 0.07)	0.14
Groups (ref. = Group 1)		
Group 2, underweight	-2.32 (-5.62, 0.97)	0.17
Group 3, underweight	-0.70 (-3.65, 2.25)	0.64
Group 4, underweight	0.33 (-3.57, 4.24)	0.87
Group 2, overweight	-0.01 (-0.44, 0.41)	0.95
Group 3, overweight	-0.03 (-0.47, 0.41)	0.90
Group 4, overweight	-0.34 (-0.92, 0.24)	0.25
Group 2, obese	-0.31 (-0.88, 0.27)	0.30
Group 3, obese	-0.04 (-0.64, 0.55)	0.89
Group 4, obese	-0.48 (-1.22, 0.26)	0.20
**DII*WC (ref. = normal)**		
Continuous DII	0.002 (-0.08, 0.09)	0.96
Groups (ref. = Group 1)		
Group 2, high risk	-0.08 (-0.55, 0.38)	0.72
Group 3, high risk	0.14 (-0.34, 0.62)	0.57
Group 4, high risk	-0.19 (-0.79, 0.40)	0.53
**DII*CMDs (ref. = CMD-free)**		
Continuous DII	0.05 (-0.10, 0.20)	0.53
Groups (ref. = Group 1)		
Group 2, CMDs	0.44 (-0.32, 1.19)	0.26
Group 3, CMDs	0.18 (-0.63, 0.99)	0.66
Group 4, CMDs	0.37 (-0.69, 1.43)	0.49

Models were adjusted for age, sex (except for DII*sex interaction models), race, education, socioeconomic status, energy intake, body mass index (except for DII*BMI interaction models), smoking status, physical activity, cardiovascular disease (except for DII*CMDs interaction models), type 2 diabetes (except for DII*CMDs interaction models), hypertension, and Alzheimer's disease polygenic risk score.

Abbreviations: BAG, brain age gap; BMI, body mass index; CI, confidence interval; CMD, cardiometabolic disease; DII, Dietary Inflammatory Index; WC, waist circumference.

References

1. Shivappa N, Steck SE, Hurley TG, Hussey JR, Hébert JR. Designing and developing a literature-derived, population-based dietary inflammatory index. *Public Health Nutr*. 2014;17(8):1689-96. doi:10.1017/s1368980013002115
2. Alfaro-Almagro F, Jenkinson M, Bangerter NK, et al. Image processing and quality control for the first 10,000 brain imaging datasets from UK Biobank. *Neuroimage.* 2018(166):400-424. doi:10.1016/j.neuroimage.2017.10.034
3. UK Biobank. UK Biobank Brain Imaging Documentation. Accessed November 15, 2022. https://biobank.ndph.ox.ac.uk/crystal/ukb/docs/brain_mri.pdf
4. Cole JH. Multimodality neuroimaging brain-age in UK biobank: Relationship to biomedical, lifestyle, and cognitive factors. *Neurobiol Aging*. 2020;92:34-42. doi:10.1016/j.neurobiolaging.2020.03.014
